# SCaMC-1Like a Member of the Mitochondrial Carrier (MC) Family Preferentially Expressed in Testis and Localized in Mitochondria and Chromatoid Body

**DOI:** 10.1371/journal.pone.0040470

**Published:** 2012-07-06

**Authors:** Ignacio Amigo, Javier Traba, Jorgina Satrústegui, Araceli del Arco

**Affiliations:** 1 Área de Bioquímica, Centro Regional de Investigaciones Biomédicas (CRIB), Facultad de Ciencias Ambientales y Bioquímica, Universidad de Castilla-La Mancha, Toledo, Spain; 2 Departamento de Biología Molecular, Centro de Biología Molecular Severo Ochoa UAM-CSIC, and CIBER de Enfermedades Raras (CIBERER), Universidad Autónoma de Madrid, Madrid, Spain; Ben-Gurion University of the Negev, Israel

## Abstract

Mitochondrial carriers (MC) form a highly conserved family involved in solute transport across the inner mitochondrial membrane in eukaryotes. In mammals, ATP-Mg/Pi carriers, SCaMCs, form the most complex subgroup with four paralogs, SCaMC-1, -2, -3 and -3L, and several splicing variants. Here, we report the tissue distribution and subcellular localization of a mammalian-specific SCaMC paralog, *4930443G12Rik/SCaMC-1Like* (*SCaMC-1L*), which displays unanticipated new features. SCaMC-1L proteins show higher amino acid substitution rates than its closest paralog SCaMC-1. In mouse, SCaMC-1L expression is restricted to male germ cells and regulated during spermatogenesis but unexpectedly its localization is not limited to mitochondrial structures. In mature spermatids SCaMC-1L is detected in the mitochondrial sheath but in previous differentiation stages appears associated to cytosolic granules which colocalize with specific markers of the chromatoid body (CB) in post-meiotic round spermatids and inter-mitochondrial cement (IMC) in spermatocytes. The origin of this atypical distribution was further investigated by transient expression in cell lines. Similarly to male germ cells, in addition to mitochondrial and cytosolic distribution, a fraction of SCaMC-1L-expressing COS-7 cells display cytosolic SCaMC-1L-aggregates which exhibit aggresomal-like features as the CB. Our results indicate that different regions of SCaMC-1L hinder its import into mitochondria and this apparently favours the formation of cytosolic aggregates in COS-7 cells. This mechanism could be also operational in male germ cells and explain the incorporation of SCaMC-1L into germinal granules.

## Introduction

The Mitochondrial carrier (MC) family (SLC25) is a highly represented group of solute transporters in eukaryotic cells. Members of MC family (MCF) perform the transport of metabolites, nucleotides and cofactors across the inner mitochondrial membrane (reviewed in [1]–[3]). The MCF shows increased complexity in pluricellular eukaryotes, mainly as a consequence of the generation of novel paralogs [3]–[6] and, to a lesser extent, by the emergence of proteins with new transport capabilities [3], [7]. Gene duplication events have contributed as the major source of MCF functional diversity [3], [5]–[8].

In mammals, the most expanded subfamilies of MCs are those involved in adenine nucleotide transport; the ADP/ATP translocases (AACs) and SCaMCs, the ATP-Mg/Pi carriers [9]–[11]. ATP-Mg/Pi carriers mediate a reversible electroneutral exchange between ATP-Mg^2−^ and HPO_4_^2−^ [12] and control the net transport of adenine nucleotides across the inner mitochondrial membrane [12]. SCaMCs belong to a MC sub-group containing N-terminal extensions with EF-hand calcium-binding motifs (CaMC, [13]). A single SCaMCs counterpart, Sal1p, is found in yeast [14], [15] while four paralogs, with several splicing variants, SCaMC-1, -2, -3 and -3L, have been identified in human and rodent genomes [6], [9], [10], [13], [16], [17]. Both subfamilies have paralogs expressed specifically in testis, SCaMC-3L and AAC4 [4], [6], [18], [19]. Interestingly, inactivation of *Aac4* in mouse causes spermatogenesis arrest [20] indicating that ATP supply from mitochondria may be critical for normal male germ cell differentiation.

Mammalian spermatogenesis is an intricate process of cellular differentiation performed by numerous testis and/or germ cell specific genes. A number of ultrastructural studies have revealed that mitochondria of germinal cells undergo changes in their appearance, location and number during spermatogenesis ([21], [22] and references therein). Additionally, mitochondria participate in the formation of the chromatoid body (CB), a male germ cell-specific perinuclear granule formed by aggregates of electron-dense material involved in the control of mRNA stability and translation, and in processing of small RNAs [23]–[27]. In mouse, the CB appears for the first time in the cytoplasm of meiotic spermatocytes in the interstices of mitochondrial clusters, named inter-mitochondrial cement (IMC) [27]–[28], being condensed after meiosis to one single perinuclear granule placed at the surface of the haploid nucleus [28]. Consequently, several mitochondrial proteins have been detected as components of CBs [29]–[31]. Furthermore, recent reports have shown that signaling from mitochondria influences the formation of the IMC and promotes mitochondrial clustering [32]–[33].

In this study, we report the tissue distribution and subcellular localization of a fifth SCaMC paralog, 4930443G12Rik/SCaMC-1Like (SCaMC-1L), expressed in male germ cells which represents a new link between mitochondria and germinal granules. SCaMC-1L is detected in the mitochondrial sheath of mature spermatids, but its intracellular location varies in a stage-specific fashion during spermatogenesis, being present in germinal granules, IMC and CB, in spermatocytes and post-meiotic spermatids. By ectopic expression in cell lines we have reproduced this complex distribution and analysed the mechanisms involved in the formation of cytosolic SCaMC-1L aggregates.

## Results

### *SCaMC-1Like* a mammalian-specific *SCaMC* paralog

**Figure 1:**
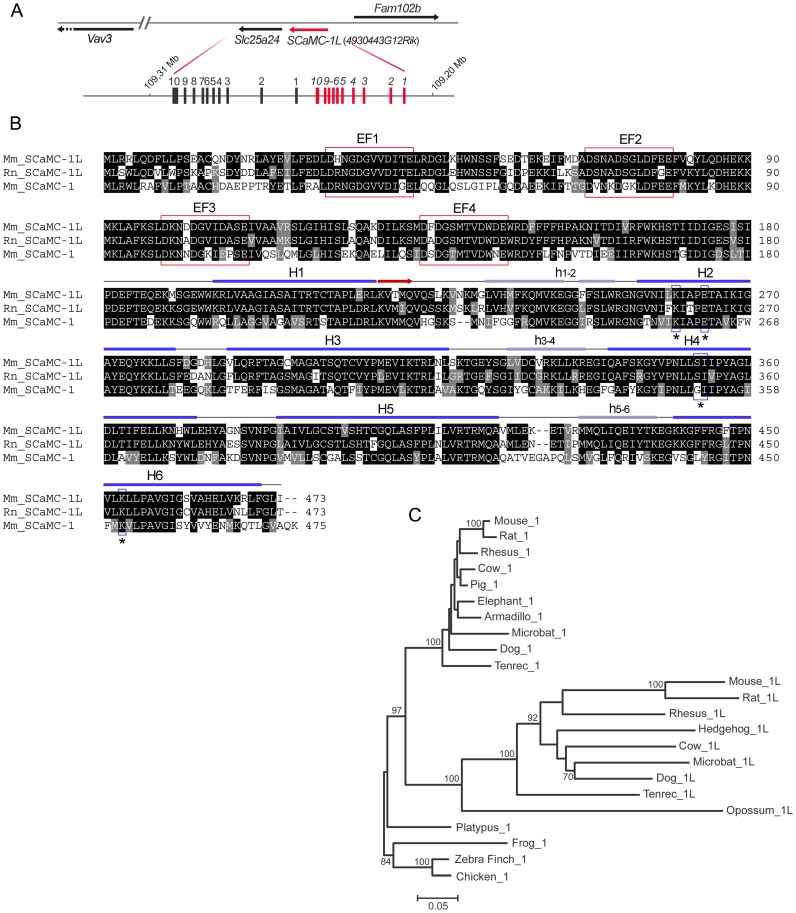
*SCaMC-1Like, SCaMC-1L*, a new SCaMC paralog emerged by a tandem duplication in mammals. (**A**) Scheme of the head-to-tail tandem array of mouse *SCaMC-1 (slc25a24)* and *4930443G12Rik/SCaMC-1L* genes. *SCaMC-1*, *SCaMC-1L* and flanking loci are represented by arrows indicating transcription orientation. *SCaMC-1*/*SCaMC-1L* intron-exon organization is also shown. Exons are indicated by filled boxes (not to scale) and numbered. (**B**) Alignment of predicted mouse and rat SCaMC-1L protein sequences (Mm_SCaMC-1L and Rn_SCaMC-1L) with that of mouse SCaMC-1 (slc25a24, Mm_SCaMC-1). Alignment was performed with ClustalW and colored with BOXSHADE 3.21 software. Predicted EF-hand calcium-binding motifs are indicated by red boxes. Secondary structure prediction for the region homologous to mitochondrial carriers, amino acids 181 to end, of Mm_SCaMC-1L was obtained using Jpred3 server [69]. The predicted transmembrane helices are indicated (H1–H6), matrix loops are marked in lower case letter and the β-strand region by an arrow. The residues proposed as participants in substrate interactions in H2, H4 and H6 [34] are included in boxes and marked with asterisks. (**C**) Phylogenetic relationships among SCaMC-1 and SCaMC-1L paralogs. The phylogenetic tree was constructed using amino acid sequences derived from exons 2 to 7 with the neighbor-joining method (MEGA 4.0, [70]) and PAM distances. Non-mammalian vertebrate SCaMCs were used as outgroups. The scale of branch lengths is indicated (number of substitutions per site). Percentage bootstrap values are shown in each node (500 replicates, only bootstrap values of 60% or more are shown). The accession numbers of annotated SCaMC-1 and SCaMC-1L proteins as well as the amino-acid sequences of manually assembled orthologs are compiled in Supplementary Tables S1 and S2. See alignment in Figure S6.

In mouse, TBLATN searches using human SCaMC-1 as query detected a predicted gene adjacent to SCaMC-1 5′ end, *4930443G12Rik*, whose product showed high similarity, 78% and 75% at the amino acid level to human and mouse SCaMC-1, respectively (SLC25A24 and slc25a24, Figure 1A). This *SCaMC-1* like gene, hereafter referred as *SCaMC-1Like* (*SCaMC-1L*), encoded a predicted protein of 473 amino acids which displayed four calcium-binding motifs at identical positions to those of mouse SCaMC-1 and a carboxyl-terminal half similar to the MCF (Figure 1B). The amino acid changes, compared to mouse SCaMC-1, were scattered throughout the protein and there was a relative abundance of conservative substitutions (Figure 1B). Its genomic organization matches that of mouse *SCaMC-1*, with 10 exons and identical exon/intron boundaries. Both genes, separated by a short intergenic region were oriented in the same direction, suggesting that they could have arisen by a tandem duplication event (Figure 1A). In the rat genome, a *SCaMC-1L* ortholog, *LOC691448*, was also found immediately upstream to *slc25a24*. *LOC691448* encoded a predicted protein of 473 amino acids, annotated as similar to slc25a24, and is 83% identical in amino acid sequence to mouse SCaMC-1L (Figure 1B). Both murine SCaMC-1L orthologs conserved the residues proposed to be involved in substrate interaction in the ATP-Mg/Pi carriers at equivalent positions (Figure 1B) [34] suggesting that SCaMC-1L could represent a ATP-Mg/Pi carrier paralog.

Searches in annotated databases failed to detect orthologs in non-mammalian vertebrates but detected *SCaMC-1L* orthologs in a wide range of mammalian clades including marsupials but not in the monotreme platypus, suggesting that duplication of the *SCaMC-1L*-ancestor occurred after the therian/monotreme split. To test this hypothesis, we compiled mammalian SCaMC-1 and SCaMC-1L orthologs (Supplementary Tables S1 and S2) and constructed a phylogenetic tree using non-mammalian SCaMC-1 proteins as basal outgroup. The topology of the tree corroborated that the ancestor duplication probably occurred in mammals after the divergence of the monotreme lineage. Thus, mammalian SCaMC-1L and SCaMC-1 orthologs formed two independent and well-defined clusters whose last common ancestor was the platypus SCaMC-1 ortholog (Figure 1C). In addition, this analysis indicated that a different rate of amino acid substitutions existed inside each cluster, with longer branches leading to SCaMC-1L than to SCaMC-1 orthologs (Figure 1C). To detect differences in the pattern of sequence substitutions between *SCaMC-1* and *-1L* genes, we calculated non-synonymous (*Ka*) and synonymous (*Ks*) nucleotide substitution rates by pairwise comparisons between representative mammalian orthologues using the codeml program implemented in the PAML package [35] (Figure S1). The *Ka*/*Ks* ratio is commonly used as indicator of selective constraints on the protein-coding genes with *Ka/Ks* = 1, <1 and >1 indicating neutral evolution, purifying and positive selection, respectively. Among *SCaMC-1* orthologs we found a very low pairwise *Ka*/*Ks* ratio, 0.055±0.012 (average ± SEM), indicative of a strong purifying selection. As anticipated, the average pairwise *Ka*/*Ks* ratio found among *SCaMC-1L* orthologs was significantly larger than this of *SCaMC-1*, 0.392±0.085 (average ± SEM; paired t-test; p<0.001) (Figure S1). This greater *Ka/Ks* ratio for *SCaMC-1L* genes was due to increased non-synonymous substitutions rate, *Ka*, which was 4.8 fold larger than for *SCaMC-1* ones (0.166±0.036 *vs* 0.034±0.007) whereas that synonymous substitutions, *Ks*, value appeared only slightly lower among *SCaMC-1L*, 0.427±0.093, than for *SCaMC-1* genes, 0.600±0.131), indicating that SCaMC-1L appears to be subjected to reduced evolutionary pressure to maintain conserved residues compared to SCaMC-1.

### SCaMC-1L is preferentially expressed in testis

**Figure 2:**
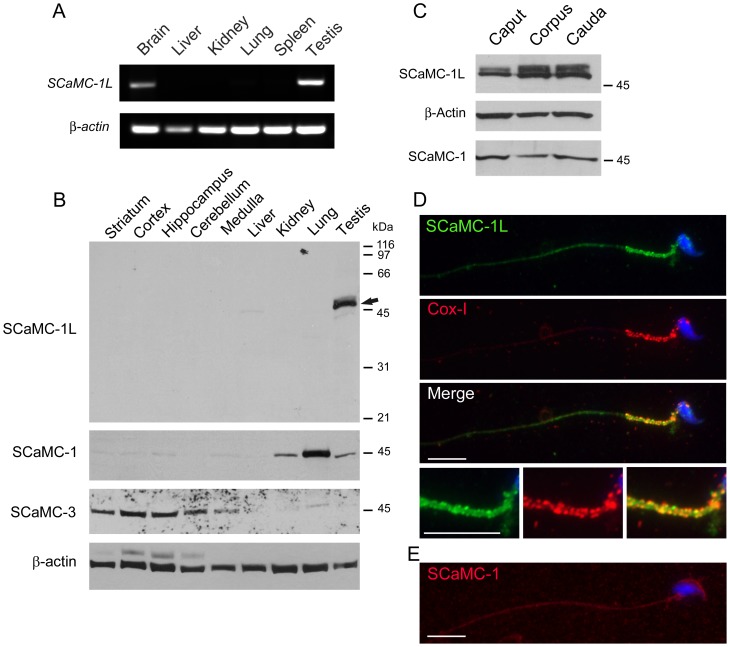
SCaMC-1L is expressed in testis and male germ cells. (**A**) RT-PCR analysis of *SCaMC-1L* expression in mouse tissues. Equivalent aliquots of cDNAs derived from the indicated tissues were used as templates. Amplification of *β-actin* was used as an internal control. The results obtained indicate that SCaMC-*1L* is expressed preferentially in testis and, at lower levels, in brain. (**B**) Expression of SCaMC-1L protein in adult mouse tissues. 10 µg of total protein extracts from the indicated tissues were analyzed by western blot using a specific anti-SCaMC-1L antibody. A single band of the expected size, around 50 kDa, was exclusively detected in testis, marked with an arrow. β-actin levels are shown as loading control. A parallel blot was incubated with antibodies against SCaMC-1 which detected a single band of 45 kDa, and then with antibodies against SCaMC-3 which detected a band of of about 48 kDa. The specific distribution patterns of the labelled bands in mouse tissues rule out any significant crossreactivity among paralogs. (**C**) 5 µg of total proteins from cauda, corpus and caput spermatids were analyzed by western blot with anti-SCaMC-1L antibody. Membranes were re-probed with β-actin antibody as loading control and anti-SCaMC-1. Equivalent SCaMC-1L levels are found in spermatids from different regions of epididymis. (**D**) SCaMC-1L-staining is detected in the midpiece of epididymal spermatids. SCaMC-1L was detected using an affinity-purified SCaMC-1L antibody and visualized with a FITC-conjugated secondary antibody, mitochondria were stained with an anti-COX-I monoclonal antibody and visualized with a Cy3-conjugated secondary antibody, nuclei were stained with Hoechst and triple merged panel is also shown. Merge panel shows the co-localization of SCaMC-1L and COX-I staining in the mitochondrial sheath of midpiece. Enlarged images of this region are shown at bottom. (**E**) SCaMC-1 is not detected in mature spermatids. SCaMC-1 staining was performed using anti-SCaMC-1 antibody at dilution 1∶200 and visualized Alexa Fluor 555 anti-rabbit as secondary antibody, nuclei were stained with Hoechst. Scale bars; 10 µm.

The acquisition of a portion of the ancestral gene functions by the newly duplicated gene or, alternatively, a more restricted expression pattern, is a frequent situation considered as a major cause for retention of duplicated genes [36]. An initial inspection of ESTs database revealed that *SCaMC-1L* transcripts derived exclusively from testis. Subsequently, we confirmed that *SCaMC-1L* transcripts were successfully amplified from testis and, to lesser levels, from brain mRNA, but not from other tissues (Figure 2A).

Western blot analysis using an antiserum raised against its hydrophilic N-extension showed a band of the predicted mass of SCaMC-1L, approx. 50 kDa, exclusively in extracts obtained from adult mouse testis (Figure 2B) and in samples from mature spermatids recovered from the caput, corpus and caudal regions of the epididymis (Figure 2C), but not in different brain regions likely due to their lower SCaMC-1L levels (Figure 2B). Figure 2D shows the localization of SCaMC-1L in epididymal spermatids where SCaMC-1L immunofluorescence was detected in the midpiece and colocalized with the mitochondrial markers COX-I (subunit I of cytochrome c oxidase, Figure 2D) and the mitochondrial dye MitoTracker Red (not shown), indicating that SCaMC-1L is present in mitochondria of mature male germ cells. SCaMC-1, it closest paralog, was not detected by immunofluorescence in these structures (Figure 2E).

### SCaMC-1L displays both cell-and intracellular-specific localizations in the developing testis

**Figure 3:**
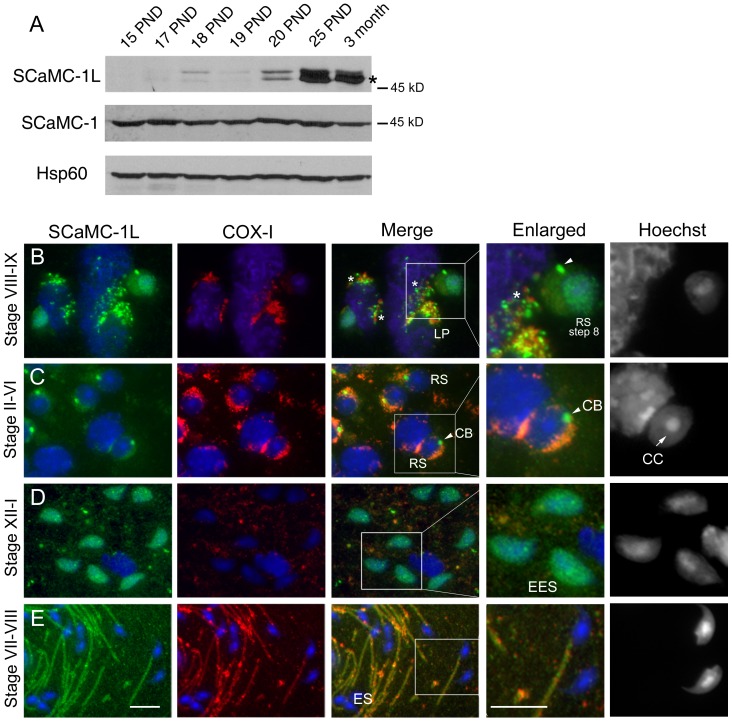
Immunohistochemical localization of SCaMC-1L protein in mouse testis. (**A**) Analysis of SCaMC-1L expression during spermatogenesis. SCaMC-1L levels were determined in mitochondrial-enriched extracts prepared from mouse testis at different post-natal days (PND), between 15–25 days and 3-month-old, by western blot. The levels of the mitochondrial proteins SCaMC-1 and hsp60 were used as loading controls. (**B–E**) Detection of SCaMC-1L in squash preparations of seminiferous tubules by immunofluorescence assays. The stages of seminiferous segments are indicated. SCaMC-1L and COX-I detection were performed as described in Figure 2. SCaMC-1L is detected in granules close to mitochondria in late pachytene spermatocytes (LP) (B), and round spermatids (RS) (B, C), identified by the heterochromatic chromocenter (CC) at the nucleus (C, indicated by an arrow). In elongating spermatids (EES) a diffuse SCaMC-1L staining is detected in the nucleus (D), in elongated spermatid (ES) SCaMC-1L is found at the midpiece matching with COX-I signals (E). Enlarged images of marked insets for merged panels and corresponding Hoechst staining are shown. Scale bars; 10 µm.

Spermatogenesis occurs in synchronized cycles within the seminiferous epithelia. In mouse, the seminiferous epithelial cycle is divided into twelve stages (I-XII) where each stage comprises a mix of cells at certain developmental steps (Figure S2, [37]). During the first wave of spermatogenesis, germ cells are synchronized in their development, and the different cell types appear progressively at different post-natal days. At postnatal day (PND) 7 only spermatogonia and Sertoli cells are present in the seminiferous tubules, primary spermatocytes appear at PND 10, while pachytene spermatocytes are not present until PND 18. After meiosis, haploid round spermatids start to differentiate to mature sperm at PND 20 [37], [38]. To study the developmental pattern of SCaMC-1L in testis, the expression of SCaMC-1L in developing male germ cells was analyzed by western blot in testis extracts at different post-natal times, between 15–25 days, and in 3-month-old mice. No significant expression was observed at PND 15 and 17, and a gradual increase in SCaMC-1L levels was observed from PND 18 to 20, reaching adult levels at PND 25 (Figure 3A). This temporal pattern indicates that SCaMC-1L expression could be limited to meiotic and post-meiotic phases of spermatogenesis (from PND 18 onwards, [38]) first appearing in pachytene spermatocytes at PND 18. The increase in SCaMC-1L levels at PND 25 corresponds to the time at which the emergence of haploid round spermatids has taken place [38]. These results are consistent with microarray data in GEO database (GEO profile GDS2390) showing that in developing testes SCaMC-1L-expression is first detected in pachytene spermatocytes and later increased in round spermatids. In early stages, at PND 18, SCaMC-1L was detected as a single faint band larger than 50 kDa, but later, at PND 19–20, an additional smaller product of approx. 50 kDa, was observed which remained as the major band at PND 25 and adult testis (Figure 3A, asterisk).

To determine its cellular distribution along spermatogenesis, we performed immunofluorescence analysis on squash preparations of seminiferous tubules as described previously [39] (Figures 3B–E). As expected by its absence in early post-natal stages, no expression of SCaMC-1L was observed in spermatogonia or Sertoli cells (not shown). SCaMC-1L was detected in meiotic spermatocytes and in all post-meiotic stages, but, unexpectedly, we found that its intracellular localization suffered dynamic changes during spermiogenesis. SCaMC-1L protein was detected in late pachytene spermatocytes (LP) at stages VII–IX of the seminiferous epithelial cycle in cytosolic granules adjacent, but not coincident, to COX-I positive mitochondria (Figure 3B, marked with asterisks). The appearance of these granular structures was similar to that described for the inter-mitochondrial cement in meiotic spermatocytes [28]. In haploid round spermatids (RS) at stages II–IV, which were identified by the presence of heterochromatic chromocenter (CC) within the nucleus, significant SCaMC-1L immunoreactivity was detected in a compact structure juxtaposed to the nuclear envelope similar to the chromatoid body (CB, arrowheads in Figure 3C). Notably, it has been proposed that CB is formed, at least in part, from the accretion of cytoplasmic granules previously aggregated in the vicinity of mitochondria in pachytene spermatocytes [27], [28], [32]. Later, after the onset of CB disappearance at step 8 of spermiogenesis, SCaMC-1L-specific signals were detected in the remains of CB and as a speckled staining spread through the cytosol and nucleus (see inset in Figure 3B, arrowhead). In successive stages, when the remainder of CB migrates to the caudal end in early elongating spermatids (EES) [28], [40], only the nuclear staining remained apparent (Figure 3D). However, in elongated spermatids (ES) at stages VII–VIII, as in mature epididymal spermatozoa, SCaMC-1L co-localized with COX-I in the mitochondrial sheath of midpiece (Figure 3E).

**Figure 4:**
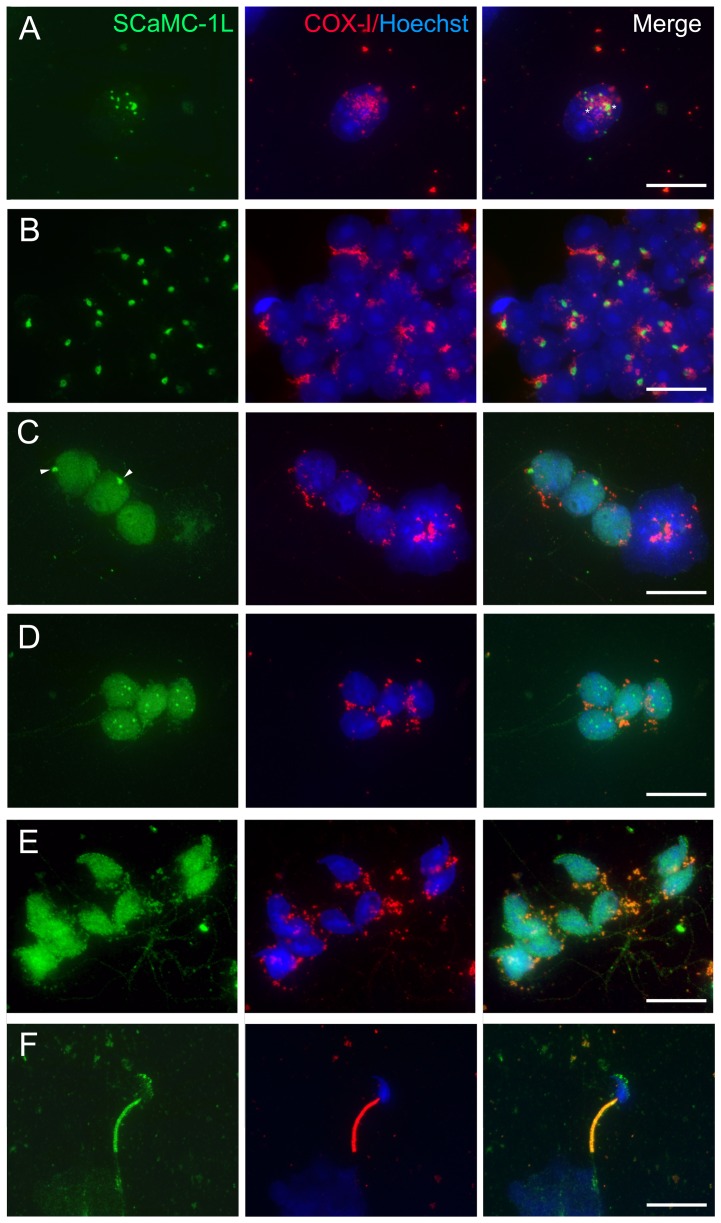
Intracellular distribution of SCaMC-1L in drying-down preparations of mouse seminiferous tubules. Immunofluorescence staining of SCaMC-1L in drying-down slides was performed as in Figure 2. Representative images of SCaMC-1L (green) and the mitochondrial marker COX-I (red) staining in late pachytene spermatocytes (**A**), haploid round spermatids (**B**), elongating spermatids at different steps of differentiation (**C**, **D**, **E**) and elongated spermatids (**F**) are shown. Scale bars; 10 µM.

**Figure 5:**
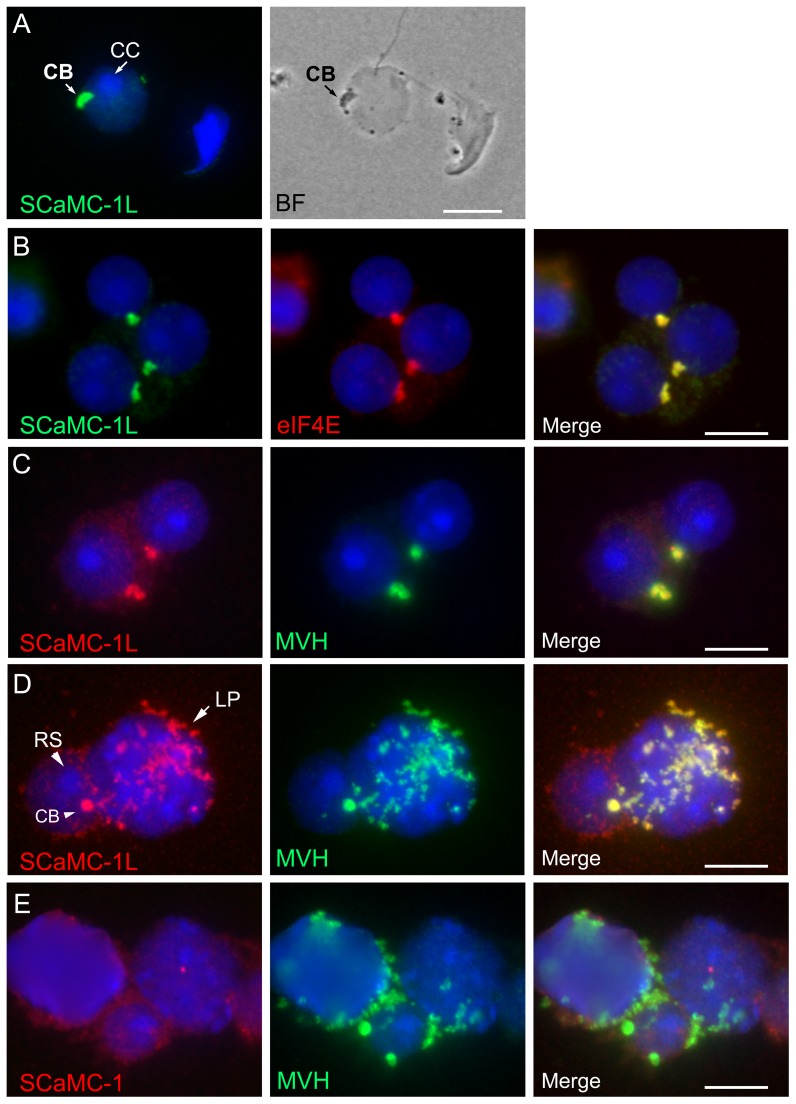
Localization of SCaMC-1L in chomatoid body. Drying-down slides were labeled with anti-SCaMC-1L antibody and its localization in chromatoid body (CB) of round spermatids (RS) was confirmed by parallel phase contrast microscopy (**A**) and by co-staining with the specific markers for CB, eIF4E (**B**) and MHV (**C, D**). CBs, identified as eIF4E and MVH protein-positive structures, appear strongly stained for SCaMC-1L. In late pachytene spermatocytes (LP) SCaMC-1L signals co-localizes entirely with cytosolic granules MVH-positive (D). RS were identified by the heterochromatic chromocenter (CC) at the nucleus (indicated by arrow in A). (**E**) Absence of co-localization of SCaMC-1 and MVH signals in drying-down slides. Scale bars; A, 5 µM; B–E; 10 µM.

In parallel, SCaMC-1L distribution was analyzed by the drying-down technique [39], [41]. Using this procedure the cytoplasm is partially lost, exposing the chromatoid body, which usually stays in contact with the nucleus along with some cytosolic organelles [39]. In these preparations, SCaMC-1L staining was also found associated to structures reminiscent of inter-mitochondrial cement in pachytene spermatocytes (Figure 4A, asterisks) and CB in round spermatids (Figure 4B). In EES, in addition to structures derived from the CB (arowheads), a diffuse SCaMC-1L staining was also detected in the nucleus (Figure 4C) which remained until the latest steps of spermatid differentiation (Figures 4D, 4E). However in ES, at the end of spermiogenesis after mitochondrial disposition around the flagellum, SCaMC-1L-positive signals appeared again restricted to the midpiece showing total co-localization with COX-I (Figure 4F). We confirmed by phase-contrast microscopy that SCaMC-1L staining was entirely coincident with the dense material associated to the nuclear envelope distinctive of CB [42] (Figure 5A). In addition, by double immunostaining we confirmed its co-localization with eIF4E, a P-body marker found in the CB [24], [43], and MVH (mouse vasa homologue) a specific marker for CB and IMC [43], [44]. As shown in Figure 5, SCaMC-1L-signals matched entirely with those of eIF4E and MVH in RS, confirming its location in the CB (Figures 5B, 5C). Likewise, in pachytene spermatocytes SCaMC-1L-staining was coincident with MVH-positive granules indicating its presence in the IMC (Figure 5D). These MVH-positives germinal granules, however, showed no colocalization with SCaMC-1 (Figure 5E).

### Ectopically expressed SCaMC-1L shows different intracellular patterns in COS-7 cells

**Figure 6:**
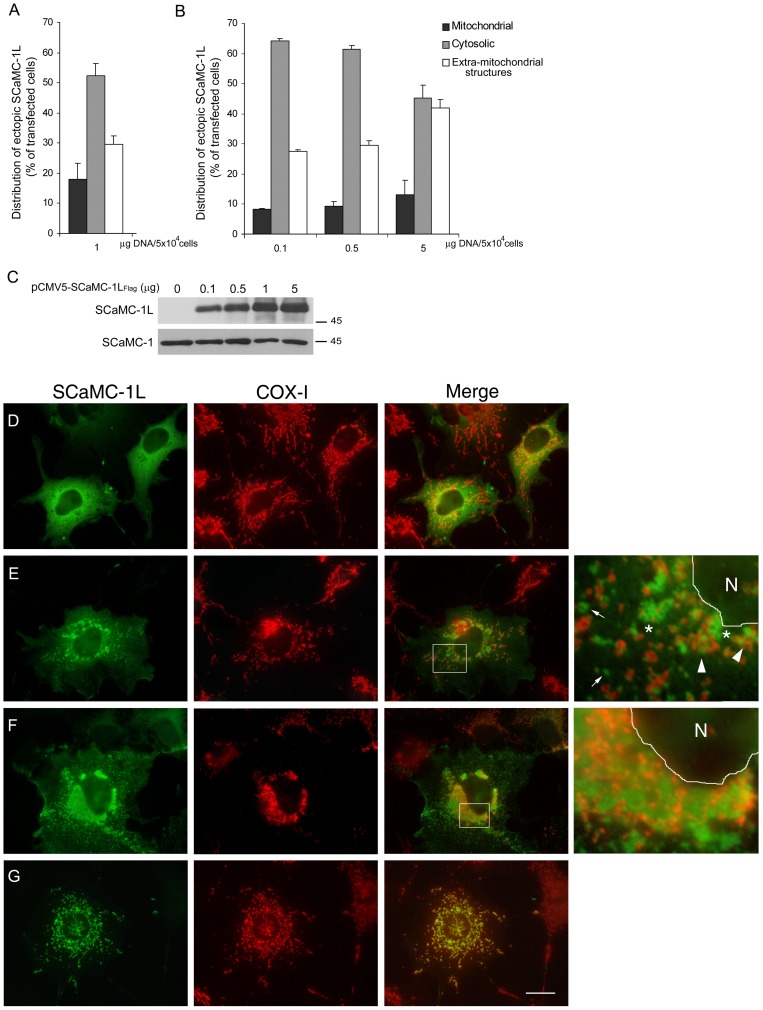
Expressed SCaMC-1L shows different intracellular patterns in COS-7 cells. Quantitative assessment of SCaMC-1L distribution in COS-7 cells. The percentage of SCaMC-1L-expressing cells showing cytosolic, mitochondrial or extra-mitochondrial patterns were analyzed using one µg of DNA (**A**) or with different DNA concentrations (**B**). Intracellular pattern was determined in parallel transfections by co-staining with anti-SCaMC-1L and anti-COX-I, as mitochondrial control, and the distribution of SCaMC-1L-signals were examined by visual inspection under a fluorescent microscope. Two hundred cells were analyzed for each transfection assay. Data are the mean ± SEM of three independent experiments. (**C**) The expression levels of SCaMC-1L at the different plasmid concentrations used were determined by western blot. Five µg of total protein was loaded in each lane. As loading control, SCaMC-1 levels was analyzed (**D–F**) Representative images of SCaMC-1L-expressing COS-7 cells showing the different intracellular patterns observed; cytosolic (D) mitochondrial (G) and disperse (E) or perinuclear SCaMC-1L extra-mitochondrial aggregates (F). Mitochondrial co-localization was determined by double staining with anti-SCaMC-1L and anti-COX-I antibodies as described in Figure 2, corresponding merge panels are also shown. Scale bar, 20 µm. Insets show higher magnification (600×) of the indicated areas in the merge panels, in both the nuclear area (N) is delimited.

The presence of MCs in non-mitochondrial membranes, as peroxisomes or plasma membrane, is not new [3], [45]. However, the dynamic distribution of SCaMC-1L during spermatogenesis and especially, its location at the CB, a non-membrane-bound compartment, were novel aspects not described for other MCs. In order to study whether this novel distribution was property of the SCaMC-1L sequence itself, we transfected cell lines lacking endogenous SCaMC-1L to analyze its intracellular distribution (Figure 6).

When a carboxyl FLAG-tagged SCaMC-1L was expressed in COS-7 cells, only a fraction of the SCaMC-1L-positive cells, about 20%, showed total co-localization of SCaMC-1L-staining with that of mitochondrial markers as COX-I (Figure 6G) or MitoTracker (not shown) (see summary in Figure 6A). However, in a large percentage of transfected cells, around 50%, SCaMC-1L appeared diffusely distributed throughout the cytoplasm (Figure 6D) and in the remaining cells, approx. 30%, in addition to cytoplamic staining, SCaMC-1L was observed concentrated in cytoplasmic granules negative for mitochondrial markers (Figure 6E, F). Similar patterns were obtained with different fixation protocols or using other cell lines, such as HEK-293T or HeLa (not shown). Likewise, identical distribution was found using construct lacking the FLAG epitope or fused to EGFP at C-teminus (Figures S3A and S3B).

Transfections with varying amounts of the SCaMC-1L-expression vector ruled out that the formation of these extra-mitochondrial granules could be an artefact due to overexpression (Figure 6B). The frequency of cells showing cytoplasmic granules was maintained when the plasmid concentration was reduced 5- or 10-fold and only using higher plasmid concentration, up to 5-fold, it increased about 15% (Figure 6B). The correlation between the expression levels of SCaMC-1L and plasmid concentration was confirmed by western analysis (Figure 6C). Therefore, ectopic expression of SCaMC-1L in cultured cells generated different intracellular patterns, as in male germ cells, in contrast to the exclusive mitochondrial location shown by its closest paralog, SCaMC-1, and by other CaMCs overexpressed in cells lines [6], [9], [10], [46], [47].

In SCaMC-1L-expressing cells containing cytoplasmic SCaMC-1L granules two major patterns were clearly distinguished; i) cells showing small, vesicular, filamentous or cup-shaped aggregates scattered throughout the cytoplasm (Figure 6E, arrows), which sometimes appeared grouped in reticular arrangements (Figure 6E, asterisks), and ii) cells showing larger and more intense SCaMC-1L-positive accumulations, which occasionally formed large honeycomb-like structures (Figure S4), located at the perinuclear area and co-clustered with mitochondria (Figure 6F). These patterns were not independent but occurred sequentially. Thus, in SCaMC-1L-positive cells disperse granules were observed early, at 6–8 h post-transfection, whereas perinuclear aggregations, showing more intense signals, emerged later, suggesting that the latter were originated by fusion of dispersed granules (movie S1). Interestingly, disperse SCaMC-1L-positive granules were usually observed very close to mitochondria, an arrangement reminiscent of the inter-mitochondrial cement (Figure 6E, arrowheads). In these cells, mitochondria appeared punctuated rather than filamentous, indicating that mitochondrial morphology could also be affected (compare COX-positive mitochondria in Figures 6E and 6F with that in 6G). In addition, we find a total absence of co-location with CD63 and Lysotracker indicating that SCaMC-1L-positive structures did not belong to the late endosomal/lysosomal pathway (Figure S5).

Taken together, our results suggest that ectopic SCaMC-1L are not successfully imported into mitochondria and it is possible that the excess of unfolded (and possibly unprocessed) cytosolic SCaMC-1L may favour the formation of small cytosolic granules that are later assembled in larger perinuclear aggregates. It should be noted that this mechanism could be also operational in male germ cells and to contribute to CB formation during spermatogenesis. Similarly, TDRD4/RNF17, a component of mammalian germinal granules, forms large cytosolic granules when is transfected in cell lines [48].

### Perinuclear SCaMC-1L-positive aggregates shows aggresomal features

**Figure 7:**
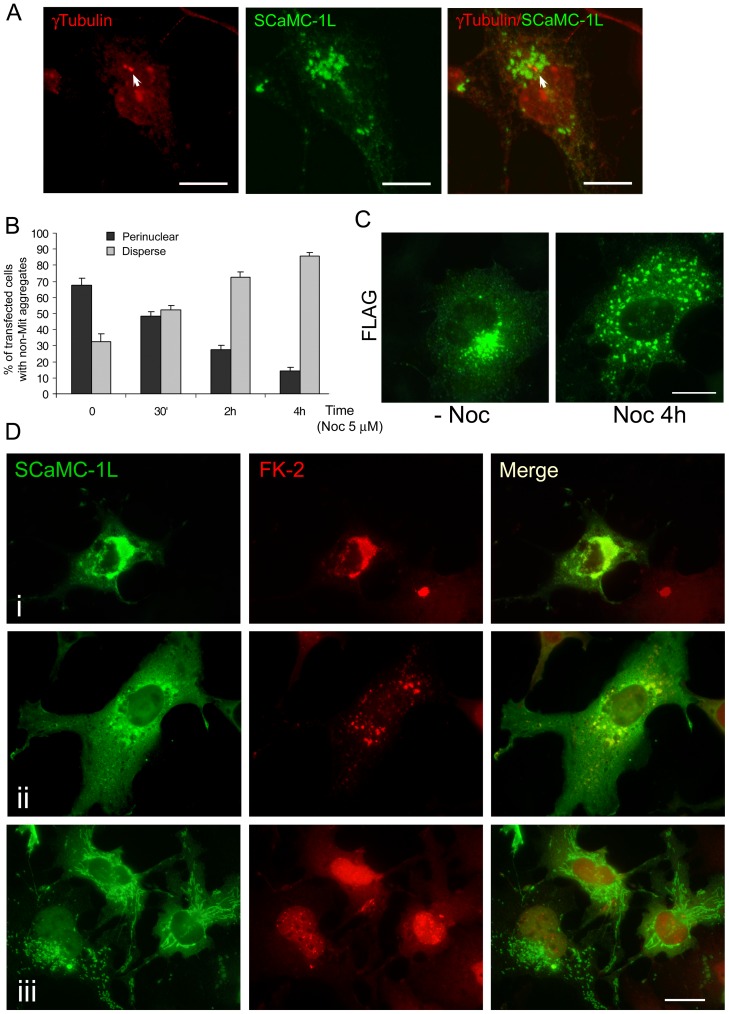
Perinuclear SCaMC-1L-positive aggregates show aggresomal features. (**A**) Perinuclear SCaMC-1L aggregates (green) colocalize with γ-Tubulin signals (red) at MTOC (indicated by an arrow). (**B**) Perinuclear SCaMC-1L aggregates formation depends on microtubule network integrity. COS-7 cells were transfected with SCaMC-1L expression vector and 24 h later incubated with 5 µM nocodazole (Noc) during the indicated time. Cells were then fixed and processed to detect SCaMC-1L distribution by immunofluorescence with anti-FLAG antibody. Only cells showing extra-mitochondrial structures were taken into account. The percentage of SCaMC-1L expressing-cells showing perinuclear and disperse aggregates at different times of Noc incubation was determined. The distribution pattern was scored in at least two hundred cells at each time point. Results are the mean ± SEM of three independent experiments. (**C**) Representative images of cells showing perinuclear (without Noc, -Noc) and dispersed (after incubation with Noc for 4 h, Noc 4 h) SCaMC-1L aggregates. Incubation with Noc only causes the dispersion of perinuclear aggregates, scattered SCaMC-1L-positive structures are still detected after the treatment. (**D**) SCaMC-1L aggregates are stained with anti-ubiquitin (FK-2) antibodies. Images of SCaMC-1L transfected cells with anti-FLAG antibody (green) showing perinuclear (i) and disperse aggregates (ii), and mitochondrial distribution (iii) and their corresponding co-staining with FK-2 (red). Merged panels are also shown. Scale bars, 20 µm.

It has been reported that the CB has aggresome-like features [26], [31]. Among other aspects, aggresomes have been characterized as inclusion bodies placed around the microtubule organizing center (MTOC) where protein aggregates were concentrated by the microtubule dynein motor [49], [50]. We observed that the large perinuclear aggregates found in SCaMC-1L-expressing cells were reminiscent of aggresomes. First, perinuclear aggregates were located in the vicinity of the MTOC, as indicated by their proximity to centrosomal γ-tubulin (Figure 7A, marked by arrow). Second, its formation depended on dynein-dependent transport, as was inferred from the loss of SCaMC-1L perinuclear aggregates upon treatment with nocodazole (Noc) (Figures 7B, 7C), a microtubule-destabilizing reagent which causes a similar disintegration of chromatoid bodies [51] (Figures 7B, 7C). Incubation with 5 µM Noc, resulted in a loss of microtubule filaments verified by α-tubulin co-staining (not shown) and caused a time-dependent decline at the percentage of SCaMC-1L-expressing cells with perinuclear aggregates, ranging from around 70%, in non-treated cells, to less than 20% after 4 hours of Noc incubation (Figure 7B) indicating that the formation of perinuclear aggregates required an unaltered microtubule network and dynein dependent transport [50]. Interestingly, small SCaMC-1L-aggregates were maintained even in the presence of Noc (Figure 7C), indicating that the disrupting agent altered their retrograde transport, but their formation did not require an intact microtubule network.

Third, SCaMC-1L perinuclear aggregates as aggresomes were highly enriched in ubiquitinated proteins. Figure 7D shows that in SCaMC-1L-expressing cells displaying SCaMC-1L aggregates with either perinuclear (Figure 7D, panel i) or disperse (Figure 7D, panel ii) pattern, SCaMC-1L-positive structures co-localized with those labeled with anti-ubiquitin FK-2 antibody, which recognizes mono- and poly-ubiquitinated proteins. This co-localization suggested that SCaMC-1L protein could be modified by ubiquitination or form complexes with ubiquitinated proteins. However, we were not able to detect ubiquitination of SCaMC-1L itself by immunoblotting, indicating that ubiquitin may modify other associated proteins. Interestingly, there was total absence of co-localization with FK-2 signals in SCaMC-1L-expressing cells in which SCaMC-1L displayed cytosolic or mitochondrial pattern (Figure 7D, panel iii), in these cells FK-2 staining was found in the nucleus, a distribution observed frequently in cells with normal proteosome activity [52], [53].

### The hydrophilic N-extension and regions of the hydrophobic C-domain of SCaMC-1L impair its import into mitochondria

**Figure 8:**
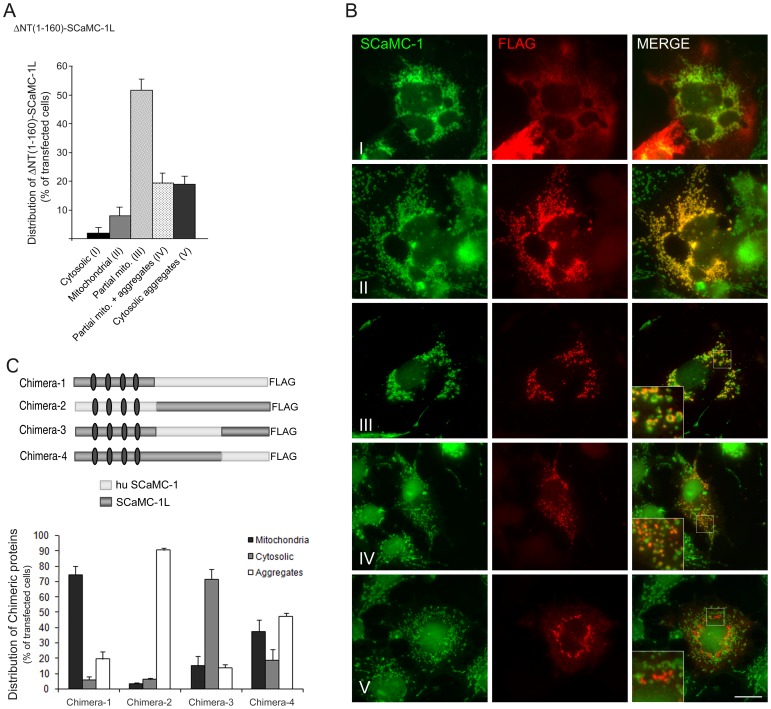
Both N-extension and C-half MC regions of SCaMC-1L are involved in its intracellular distribution. (**A**) The N-terminal extension of SCaMC-1L hampers its import into mitochondria. COS-7 cells were transfected with a FLAG-tagged amino truncated SCaMC-1L protein, ΔNT_(1–160)_-SCaMC-1L_FLAG_, and 24–30 h later were fixed and co-stained with anti-FLAG (red) and anti-SCaMC-1 (green) antibodies. FLAG and SCaMC-1 images were taken under identical conditions and co-localization was evaluated in merged compositions. Five patterns were clearly identified (I–V). Most of ΔNT_(1–160)_-SCaMC-1L-positive cells show, at distinct degree, mitochondrial localization. These cells were sub-classified according to their co-localization degree with mitochondria (II–III) and the additional presence of SCaMC-1L aggregates (IV). Results are the mean ± SEM of three independent experiments. At least 50 cells were analyzed for each transfection assay. (**B**) Representative images of FLAG/SCaMC-1 double stained cells showing ΔNT_(1–160)_-SCaMC-1L-patterns I, II, III, IV and V merged panels are also shown. Insets magnification 300×; scale bar, 20 µM. (**C**) Scheme of the chimeric SCaMC-1L/SCaMC-1 proteins used. Relative positions of the EF-hand calcium-binding domains are marked by gray ovals. Quantification of the intracellular patterns observed for each protein (bottom) was performed as described in Figure 6. One hundred cells were counted for each transfection assay. Results are the mean ± SEM of three independent experiments.

In CaMCs, as in MCs, mitochondrial targeting information is scattered along the hydrophobic C-half domain [13], [54], [55], but a significant role for the long N-terminal extension has been proposed [16]–[56]. Thus, some CaMCs show incomplete mitochondrial import that is overcome by removing its N-terminal domain [16]–[56]. We have thus analyzed whether this domain was responsible for the decreased mitochondrial import of SCaMC-1L. To this end, we generated a truncated carboxy-FLAG tagged SCaMC-1L protein lacking the entire hydrophilic N-extension (ΔNT_(1–160)_-SCaMC-1L, Figure 8A), and studied its intracellular distribution. Transiently transfected COS-7 cells showed a higher percentage of cells displaying mitochondrial anti-FLAG staining, about 80% (Figure 8A), indicating that the N-domain of SCaMC-1L affected negatively its import in mitochondria. However, only in a fraction of ΔNT_(1–160)_-SCaMC-1L expressing cells, about 10%, anti-FLAG signals entirely co-localized with mitochondrial markers (Figure 8B, panel ii); most ΔNT_(1–160)_-SCaMC-1L-positive signals showed a partial overlap with mitochondrial SCaMC-1 (Figure 8A, see Figure 8B panels iii and iv).

However, regardless of its higher frequency of mitochondrial location, ΔNT_(1–160)_-SCaMC-1L still appeared in extra-mitochondrial aggregates, both disperse or perinuclear (Figures 8A and 8B, panel v), indicating that the hydrophilic N-domain hindered the import into mitochondria but did not determine the formation of cytosolic aggregates. To study the role of the hydrophobic carboxy-terminal half of SCaMC-1L, we constructed chimeric proteins containing portions from SCaMC-1L and from human SCaMC-1 (Figure 8C). When expressed in COS-7 cells, we found that the C-terminal half of SCaMC-1 (Chimera-1, 1L_1–184_-huSCaMC-1_185–476_) conferred a predominantly mitochondrial distribution, while the C-terminal half of SCaMC-1L (Chimera-2, hu1_1–184_-SCaMC-1L_185–473_) caused a frequent localization in non-mitochondrial aggregates (more than 80%, Figure 8C). Moreover, the frequency of non-mitochondrial aggregates diminished with the length of SCaMC-1L C-half in the chimera (Figure 8C). In addition, distal and proximal regions of the C-terminal half did not affect equally to its intracellular distribution. Thus, residues 185–362 of SCaMC-1L present in Chimera-4 were more prone to promote the formation of cytosolic aggregates than the region covering the two last transmembrane domains, residues 363 to C-end, which favored its presence in mitochondria (Figure 8C). Taken together, these results suggest that both the hydrophilic N-extension and the most distal C-end regions of SCaMC-1L prevent its targeting to mitochondria while the proximal C-domain, residues 185–362, might provide aggregation-prone features to SCaMC-1L.

## Discussion

### Localization of SCaMC-1L in mouse testis

In the present study, we describe a mammalian-specific SCaMC paralog, SCaMC-1L, which displays notable differences with other MCs. SCaMC-1L expression was restricted to male germ cells and during spermatogenesis it showed a surprising number of intracellular locations which were displayed in a stage-specific fashion throughout the male germ-cell differentiation process. In mature spermatozoa the location of SCaMC-1L was that expected for a mitochondrial protein, the mitochondrial sheath (Figure 2D). However, in earlier differentiation stages, SCaMC-1L was already expressed, but not in mitochondria. In late pachytene spermatocytes, SCaMC-1L was present in MVH-positive cytosolic granules, the intermitochondrial cement (IMC, Figures 3B, 4A, 5D) [27], [28], [43], [44], and later, at early post-meiotic stages, it appeared in the cytosol and co-localized with markers of the chromatoid body (CB) (Figures 3C, 4B and 5), a unique structure in male germ cells composed by aggregates of electron-dense material and involved in RNA processing [23], [25]–[27].

A dynamic localization during spermatogenesis has also been reported for other mitochondrial proteins such as cytochrome c [29], the phospholipid hydroperoxide glutathione peroxidase, PHGPx [30] or mouse gametogenetin-binding Protein 1 (GGNBP1, [22]). Rat cytochrome c paralogs, both testis-specific, c_t_, and somatic, c_s_, have been found both in mitochondria and CB during spermatogenesis [29] and other mitochondrial proteins, such as ATP synthase subunits α and β, and proteins encoded by the mitochondrial genome, as COX-I, have been detected in the CB [31]. In addition to MVH, a number of CB components, including tudor-domain containing 1, 6 and 7 (TDRD1, TDRD6 and TDRD7), or Sm proteins of spliceosomal snRNPs have also been localized in the IMC of spermatocytes [27], [57], [58] revealing the existence of a functional interplay between the CB and mitochondria during spermatogenesis [27], in which SCaMC-1L may also participate.

Other MCs such as ORC2/slc25a2, SCaMC-3L/slc25a41 or AAC4/slc25a31 are also preferentially expressed in mouse testis [4], [6], [18], [59], but their location at the cellular level along spermatogenesis is still uncertain. Surprisingly, human AAC4/SLC25A31 has been detected in the fibrous sheath of the flagellum of spermatozoa, a portion that lacks mitochondria [19].

### Determinants of the subcellular distribution of SCaMC-1L

Our data indicate that the anomalous distribution of SCaMC-1L in male germ cells was a property depending on the sequence of SCaMC-1L itself. Ectopic expression of SCaMC-1L in cell lines revealed that SCaMC-1L had a difficulty in mitochondrial import. This was not due to artefacts of overexpression, as it was observed even when it was expressed at very low levels (Figures 6A, 6B). We analyzed in more detail the determinants for the lack of mitochondrial localization of SCaMC-1L and found that mitochondrial import of SCaMC-1L was partially hampered by its hydrophilic N-terminal extension (Figures 8A, 8B). A reversal of incomplete mitochondrial import by N-terminal domain removal has been also described for other CaMCs [16], [56]. Roesch and coworkers [56] have suggested that CaMCs interact in their pass across the intermembrane space with small Tim proteins different from those used by MCs lacking hydrophilic N-extensions, and this could account the different effectiveness observed in their import. Moreover, the faulty import of SCaMC-1L is not caused solely by its N-extension, as amino acids 185–362 within the hydrophobic SCaMC-1L carboxyl-half also impaired its import in mitochondria (Figure 8C). We can not rule out that SCaMC-1L may require additional factors to be inserted into mitochondria. Thus, it has been reported that apocytochrome c_T_ is less readily taken up by mitochondria *in vitro* than the c_S_ isoform and that specific stage-specific factors may be required to facilitate its import into mitochondria [29].

Our findings suggest that the apparent difficulty of SCaMC-1L to be imported, along with its expression levels, are important factors causing the formation of cytosolic aggregates. In fact, inefficient translocation through the IMS and slow passage across the TOM complex in the outer mitochondrial membrane favor aggregation of mitochondrial precursor proteins [54], [60], and mistargeting of overexpressed hydrophobic proteins also leads to the formation of stable cytosolic aggregates [49]. Likewise, we observed that high SCaMC-1L expression, obtained by a 5-fold increase in the amount of expression vector (Figure 6B) or longer times (48–72 h) after transfection (not shown), resulted in a higher frequency of cells showing cytosolic aggregates. This was reminiscent of the events occurring during spermatogenesis, where a large increase of SCaMC-1L expression occurred at the time of round spermatids emergence, and strong SCaMC-1L immunoreactivity appeared associated with the CBs in these cells (Figures 3C, 4B and 5).

### Physiologial role(s)

Based on sequence conservation, mitochondrial SCaMC-1L is probably a mitochondrial ATP-Mg/Pi carrier. In fact, murine SCaMC-1L orthologs show high degree of homology with SCaMC-1, including the residues proposed to be involved in substrate interaction (Figure 1B, [34]). However, its transporter function has not been investigated. SCaMC-1L adds to the large group of mitochondrial proteins having testis-specific isoforms, including cytochrome c_T_, [61], mammalian testis-specific cytochrome oxidase subunit VIb [62] and others [4], [63].

A most remarkable aspect of SCaMC-1L is its location at the CB. In addition to RNA-binding proteins and TDRD proteins, the CB contains proteins from different cellular compartments as histones, actin, glycolytic (LDH, [31]) and mitochondrial (cytochrome c_T_, [29]) proteins as well as aggresomal markers, such as Hsp70, ubiquitin and ubiquitin-conjugating enzymes [26], [31], suggesting an aggresome-like role for this structure [26], [31]. In SCaMC-1L expressing cells, perinuclear SCaMC-1L aggregates exhibit some of the hallmarks described for cellular aggresomes namely as microtubule-dependent formation, co-location with γ-tubulin at the MTOC and the presence of anti-ubiquitin positive signals (Figure 7D) [50], [64], indicating that these aggregates mimic SCaMC-1L behavior in male germ cells. Aggresomes are not only stress responses to misfolded proteins. “Physiological aggresomes” have been shown to play a role in inactivation of iNOS [65], or in the regulation of cardiomyocyte cell cycle by cyclin D2SV [66]. It is possible that during spermatogenesis, SCaMC-1L could favor the recruitment or co-aggregation of components from mitochondria or other subcellular origins by transporting them to the CB.

Another possibility is that SCaMC-1L through its Ca^2+^-binding domains can participate in the enrichment of calcium ions observed in the CB [26]. A similar function has been proposed for the Ca^2+^-calmodulin-binding proteins BASP1 and MARCKS recently identified as CB components [67]. Alternatively, SCaMC-1L could be also a structural component of the CB. In transiently transfected cells, perinuclear SCaMC-1L aggregates do not form a compact structure but fibrous aggregates as occurs for the CB (Figure S3, [28]). Interestingly, in primates (human and chimpanzee), where *SCaMC-1L* counterparts have undergone rearrangement becoming non functional, the CB appears to be formed by small particles dispersed in the cytoplasm instead of one single and large aggregate [27]. Human *SCaMC-1L*, LOC727941, is pseudogenized due to a nonsense mutation in exon 6 and the lack of sequences corresponding to mouse exons 2 and 3. In chimpanzee genome we have detected a constitutional deletion affecting exons 5–10 of the *SCaMC-1L* counterpart.

On the other hand, during post-meiotic male germ cell differentiation, transcription ceases whereas translation is delayed, and consequently, it is possible that SCaMC-1L could be transiently accumulated in the CB until its inclusion in the mitochondria of spermatids. Similarly, after CB disassembly, some CB constituents migrate appearing in mature spermatids associated to different structures of the middle piece of flagellum [40], [67].

### Evolutionary aspects

The finding of this fifth SCaMC paralog makes the ATP-Mg/Pi carriers the most represented MC subfamily in mammals. Only the ADP/ATP translocases have undergone a similar expansion [11]. Although murine SCaMC-1L shows high similarity with its closest paralog SCaMC-1, around 75% at the amino acid level (Figure 1B), there is a higher rate of amino-acid divergence (Figure 1C), due to a higher rates non-synonymous (*Ka*) nucleotide substitution rates, about 4.8 fold, in mammalian SCaMC-1L than in their corresponding SCaMC-1 paralogs. This agrees with the accelerated divergence commonly observed in one of the copies of duplicated gene pairs [68]. It is reasonable to assume that the accelerated divergence detected in SCaMC-1L paralogs enabled the acquisition of new properties, maybe related to its aggregation-prone features, advantageous for mammalian spermatogenesis.

## Materials and Methods

### *In silico* searches of SCaMC-1 and SCaMC-1Like sequences

To search for *SCaMC-1* related genes, we have used protein and nucleotide sequences of identified SCaMC-1 genes [10] to screen genome databases using the BLASTN, BLASTP, TBLASTN and BLAT algorithms. We used NCBI (http://blast.ncbi.nlm.nih.gov), Ensembl (www.ensembl.org) and UCSC (http://genome.ucsc.edu). Searches were carried out using default values with the low complexity filter off. Likewise, BLASTN were performed in genomic TRACE archives in NCBI. Sequences derived from the most closely related phylogenetic species were used as query in each search. Protein homology was determined using the BLASTP algorithm. Exon/intron boundaries were confirmed by application of the Splice site prediction by Neural Network program at BDGP (Berkeley *Drosophila* Genome Project, http://www.fruitfly.org). Finally, sequences were aligned by ClustalW followed by manual adjustments. To estimate non-synonymous (*Ka*) and synonymous (*Ks*) nucleotide substitution ratio representative mammalian mRNA sequences of SCaMC-1 and SCaMC-1L genes were retrieved from the NCBI database, and then converted to codon alignments by the PAL2NAL server (http://www.bork.embl.de/pal2nal) using corresponding protein sequence alignments. *Ka* and *Ks* values were calculated with the codeml program implemented in the PAML package [35]. Pair Student's t-test was used for statistical analysis.

### Squash and drying-down preparations of seminiferous tubules and spermatozoa processing

Tissue samples from C57BL/6 mice were used for all analysis. All animal work performed in this study was carried out in accordance with procedures approved in the Directive 86/609/EEC of the European Union with approval of the Ethics Committee of the Universidad Autónoma de Madrid. Animals were sacrificed by cervical dislocation, and the testes from adult mouse were dissected and decapsulated in phosphate buffered saline (PBS). After identification of the waves of the seminiferous epithelium by transillumination, stage-specific short tubule segments were cut and processed as described [39]. Briefly, the tubule segments were transferred to microscope slides in 15 µl of PBS. A coverslip was placed carefully onto the tubule segment, the excess fluid was removed by blotting, the slides were snap frozen in liquid nitrogen and the cover slip was removed. Cells were fixed by incubating in 90% ethanol for 3 minutes and air dried at RT.

For mouse drying-down preparations, stage-specific segments of seminiferous tubules were isolated and transferred in 20 µl of 100 mM sucrose solution to a small petri dish. Cells were released from tubules by squeezing carefully with fine forceps, and suspended by gentle pipetting up and down. The cell suspension was spread on a slide dipped in the fixing solution, (1% paraformaldehyde (PFA), 0.15% Triton X-100, pH 9.2 in PBS), and slides were dried for 3 h at 37°C in a highly humidified chamber protected from light [41]. The stage of the seminiferous epithelial cycle was determined based on the appearance of Hoechst-stained germ cell nuclei. The criteria used for staging were the presence of specific germ cell types and the combination of cell types detected.

Spermatozoa collected from dissected caput, corpus and cauda epididymis were washed in PBS, centrifuged at 3000 g and fixed in 4% PFA in PBS for 30 min at RT. Then cells were collected by centrifugation and diluted in PBS for slide preparation. Slides were dried at RT and stored at −80°C.

### RT-PCR analysis of mouse SCaMC-1-Like expression

The procedures used for total RNA extractions and first strand cDNA synthesis have been described previously [6], [10]. Amplification of mouse SCaMC-1L mRNA has been performed basically as described [6] using specific primers derived from the predicted mouse *4930443G12Rik* gene (forward, 5′-GGTCGATGACAGTAGACTGG-3′; reverse, 5′-GAGATCTAAGCCTGCGTACG-3′). For normalization, β-actin sequences were amplified in parallel. The primers and conditions used have been previously described [6].

### Plasmids

The SCaMC-1Like full-length cDNA was synthesized from 3 µg of total RNA of mouse testis with the Cells-to-cDNA™ II Kit (Ambion) using random primers to synthesize the first-strand cDNA. PCR reaction was performed using specific primers designed according to the predicted *4930443G12Rik* gene. We used two sets of PCR primers to generate overlapping fragments: 5′ pair (forward, containing ATG, 5′-CTGGACGATGCTACGCAGGC-3′and reverse 5′-TCGGTATAGGGCTCGGGTAC-3′); and the 3′ pair (forward 5′-CAACCTGCTGAGCATCATACCG-3′ and reverse 5′-TTCCACTTCTAGATTAATCCAAAAAGCC-3′). PCR fragments were cloned using pSTBlue1-blunt vector (Novagen) and sequenced. Finally, full length coding sequence was assembled and subcloned into the expression vector pCMV5 digested with EcoRI, to obtain pCMV5-SCaMC-1L. The plasmid expressing SCaMC-1L_FLAG_ was generated by using PCR to insert DNA encoding the FLAG epitope preceding the termination codon. The SCaMC-1 amino-truncated version, pCMV5-NT_(1–160)_-SCaMC-1L (amino acids 161–473), was generated by PCR using a forward primer containing a new initiation codon (underlined) and additional nucleotides according to rules for consensus for translational initiation, primer 5′-GACCATGGACATTGTTCGTTTCTGG-3′.

To obtain chimeric proteins containing regions of human SCaMC-1 and mouse SCaMC-1L, a novel EcoRI site was created into SCaMC-1L cDNA, SCaMC-1L*, at equivalent position to that present in human SCaMC-1 cDNA by PCR, this change results in a silence mutation. Chimera-1 (huN_(1–184)_-SCaMC-1L_(185–473)FLAG_) and Chimera-2 (1LN_(1–184)_-huSCaMC-1_(185–477)FLAG_) were generated exchanging EcoRI fragments between SCaMC-1L*_FLAG_ and pCMV5-SCaMC-1_FLAG_. Constructs encoding Chimera-3 (1LN_(1–184)_-huSCaMC-1_(185–362)_-1L_(363–473)FLAG_) and Chimera-4 (1LN_(1–163)_-huSCaMC-1_(364–477)FLAG_) proteins were obtained from Chimera-1 and SCaMC-1L_FLAG_, respectively, exchanging an equivalent BglII-EcoRI fragment with SCaMC-1L_FLAG_ to obtain Chimera-3 and with SCaMC-1_FLAG_ for Chimera-4.

### Antibody generation

A polyclonal antibody was raised in rabbit against bacterially expressed N-terminal extension of mouse SCaMC-1L. pQE vectors (Qiagen) were used to express amino acids 2–162 fused to a 6-histidine tag at the N terminus. Procedures for overexpression and purification of the recombinant protein were as described previously [46]. The antiserum was affinity-purified by incubation with nitrocellulose membrane strips to which the antigen was bound, followed by elution with 0.1 M glycine (pH 2.8).

### Cell culture, transfection and immunofluorescence analysis

COS-7 cells obtained from ATCC (Manassas, VA) were cultured in Dulbecco's modified Eagle's medium supplemented with 5% inactivated FBS (Invitrogen) at 37°C in a 5% CO_2_ atmosphere. For transfections, 25,000 cells grown on coverslips were transiently transfected with 0.5 µg of DNA, except when noted, using the LipofectAMINE reagent as described [16]. After 24–48 h of incubation to allow expression, cells were fixed and immunofluorescence assays performed as described [10], [46]. Identical procedures and antibodies dilutions were used for male germ cells preparations. In immunofluorescence analysis monoclonal antibodies against FLAG peptide, clone M2 (1∶100; Sigma), COX-I (1∶200; Molecular Probes), γ-tubulin (1∶200; Sigma), ubiquitinated proteins FK2 (1∶200; BIOMOL), anti-eIF4E (1∶100; Santa Cruz), anti-α-actin (1∶200; Sigma) were used, as well as rabbit polyclonal antibodies against human SCaMC-1 (1∶200; [10]), affinity-purified rabbit anti-SCaMC-1L (1∶200) and anti-MVH (1∶100; Abcam). Secondary antibodies used were Alexa Fluor 488 goat anti-rabbit (1∶400; Invitrogene); Cy3-conjugated goat-anti-mouse (1∶1000; Jackson InmunoResearch), FITC-conjugated goat-anti-mouse (1∶400; Vector) and Alexa Fluor 555 donkey anti-rabbit (1∶500; Invitrogene). For double staining anti-MVH was labeled using Zenon Rabbit IgG Labeling Kit (Invitrogen) according to manufacturer's instruction.Nuclear staining was performed with Hoechst 33258 or DAPI (Molecular Probes). Fluorescence microscopy was performed using an Axioskop2 plus (Zeiss) at a nominal magnification of 63×. Digital images were taken in parallel with a Coolsnap FX camera controlled with MetaView software.

### Subcellular fractionation and Western blot analysis

Testes were collected from mice of different ages and processed in parallel. Samples were homogenized in a dounce homogenizer in 0.8 ml of buffer A (250 mM sucrose, 20 mM HEPES, 10 mM KCl, 1.5 mM MgCl_2_, 1 mM EDTA, 1 mM EGTA, 1 mM dithiothreitol pH 7.4, 1 mM iodoacetate and 1 mM phenylmethylsulfonyl fluoride). Homogenates were centrifuged at 500 g for 15 min, and the resulting supernatants were further centrifuged at 9000 g for 15 min to obtain mitochondria-enriched fractions, which were resuspended in buffer A. Protein determination was performed according to the Bradford method. Total protein extraction from cultured cells was performed as described [10]. Samples were resolved by SDS-PAGE on a 10% gel and the presence of SCaMC-1L was determined by Western blotting using anti-SCaMC-1L (1∶3000). Rabbit anti-human SCaMC-1 and anti-human SCaMC-3 (1∶5000; [10]), and mouse monoclonal anti-hsp60 (1∶10000; Stressgene) and anti-β-actin (1∶10000; SIGMA) were used as loading control. Proteins were detected using ECL (Amersham Biosciences).

## Supporting Information

Figure S1 ***Ka/Ks*** **ratios among mammalian SCaMC-1 and SCaMC-1L orthologues.** To calculate *Ka*/*Ks* values for *SCaMC-1* and *SCaMC-1L* genes we retrieved coding sequences corresponding to exons 2 to 7 of representative mammalian and performed pairwise sequence comparisons. The results are based on Ka/Ks values for sequences from seven representative mammals; *Bos Taurus*, *Echinops telfairi*, *Myotis lucifugus*, *Mus musculus*, *Macaca mulatta*, *Pongo pygmaeus*, and *Rattus novergicus*. Ka/Ks ratios were estimated using PAML for all pairwise combinations. The pairwise Ka/Ks ratios for *SCaMC-1L* orthologs are significantly greater than those of SCaMC-1 (paired t-test; p<0.001) suggesting that *SCaMC-1L* genes evolved faster than *SCaMC-1*.
(TIF)

Figure S2 **Expression of SCaMC-1L throughout the spermatogenic stages.** The germ cells present at each stage (I–XII) of the mouse spermatogenic cycle from intermediate spermatogonia (In) to mature sperm are pictured (adapted from [37]). Each stage of the spermatogenic cycle comprises a set of developing germ cells. In the diagram, the developmental progression of a cell is followed from the bottom file, left to right. By solid colored bars are indicated the developmental stages showing significant SCaMC-1L expression, dashed bars indicate less abundant but detectable expression. The intracellular patterns detected for SCaMC-1L; intermitochondrial cement (IMC), chromatoid body (CB) and mitochondrial sheath, are marked by different colors. SCaMC-1L expression is absent in intermediate and type-B spermatogonia (B), and preleptotene (Pl), leptotene (L) and zygotene spermatocytes (Z). SCaMC-1L is observed in the IMC in late pachytene spermatocytes (P), at differentiation stage VII. After meiosis (m), SCaMC-1L is detected in the CB during round spermatid differentiation (steps 1–8 of spermiogenesis) and as dispersed structures after CB dissociation (step 9 onwards). In elongated spermatids, steps 15 and 16 of spermiogenesis, the protein is found in the mitochondrial sheath of the flagellum.
(TIF)

Figure S3 **The C-terminal does not affect the intracellular patterns of ScaMC-1L.** (**A**) Representative images of SCaMC-1L-expressing COS-7 cells showing the different intracellular patterns observed; cytosolic (a), mitochondrial (b) and extra-mitochondrial aggregates (c). COS-7 cells were transfected with a full-length SCaMC-1L construct unchanged at C-end, co-localization with mitochondrial structures was determined by co-staining with anti-SCaMC-1L and anti-COX-I as mitochondrial control. (**B**) Representative images of COS-7 cells expressing a SCaMC-1L-EGFP fusion protein. SCaMC-1L-EGFP construct was obtained subcloning the entire mouse *SCaMC-1L* coding sequence (amino acids 1–473) into pEGFP-N1. SCaMC-1L-EGFP expressing cells were fixed and co-localization with mitochondrial COX-I was determined. Cells showing cytosolic (a), mitochondrial (a) and extra-mitochondrial aggregates (b) are shown. Magnification 63×; scale bar, 10 µm.
(TIF)

Figure S4 **Perinuclear honeycomb-like SCaMC-1L aggregates.** A representative image of SCaMC-1L-transfected COS-7 cells containing SCaMC-1L honeycomb-like aggregates is shown. SCaMC-1L was detected with specific anti-SCaMC-1L antibody visualized with a FITC-conjugated secondary antibody, mitochondrial structures are stained with anti-COX-I antibody visualized with Cy3-conjugated secondary antibody; the corresponding merged panel is also shown. Magnification 63×; scale bar, 20 µm. Enlarged image (400×) of the indicated inset is also shown.
(TIF)

Figure S5 **Cytosolic SCaMC-1L granules do not co-localize with late endosomal/lysosomal markers.** COS-7 cells were transiently transfected with FLAG-tagged SCaMC-1L and 24–30 hours later co-localization was analyzed by co-staining with specific markers. COS-7 cells were co-stained with anti-SCaMC-1L antibody (A, B) and specific markers for late endosomes (A) and lysosomes (B). As late endosomal marker a monoclonal anti-human CD63 antibody (clone H5C6, Developmental Studies Hybridoma Bank) was used at 1∶200. To analyze co-localization with lysosomes, cells were stained with 50 mM LysoTracker (Molecular Probes) for 30 min at 37°C prior to fixation. Nuclei were stained with Hoechst. Magnification 63×; scale bar, 20 µm.
(TIF)

Figure S6 **Alignment of SCaMC-1 and SCaMC-1L sequences encoded by exons 2 to 7 was performed with ClustalW program and coloured using BOXSHADE program.** (www.ch.embnet.org).
(TIF)

Table S1 **Accession numbers of annotated SCaMC-1 and SCaMC-1L proteins.** (alphabetical order of species).
(DOCX)

Table S2 **SCaMC-1 and SCaMC-1L non-annotated protein sequences used in this study.** (alphabetical order of species).
(DOCX)

Movie S1 **COS-7 cells were grown on glass plates and transiently transfected with SCaMC-1L-EGFP construct.** The SCaMC-1L-EGFP expressing vector was obtained subcloning the full coding sequence (amino acids1–473) of mouse SCaMC-1L-1L into pEGFP-N. Images were taken every 15 min for 7 h using an Axiovert 200 (Zeiss) system at 37 C with a ×40 objective using a Coolsnap FX CCD camera (Roper Scientific). The Metamorph 6.1r6 (Universal Imaging) program was used for image processing.
(AVI)
