# Supplementary figures and images for: SCaMC-1Like a Member of the Mitochondrial Carrier (MC) Family Preferentially Expressed in Testis and Localized in Mitochondria and Chromatoid Body
Source: PLoS One. 2012 Jul 6;7(7):e40470. doi: 10.1371/journal.pone.0040470 (PMC3391283; doi:10.1371/journal.pone.0040470)

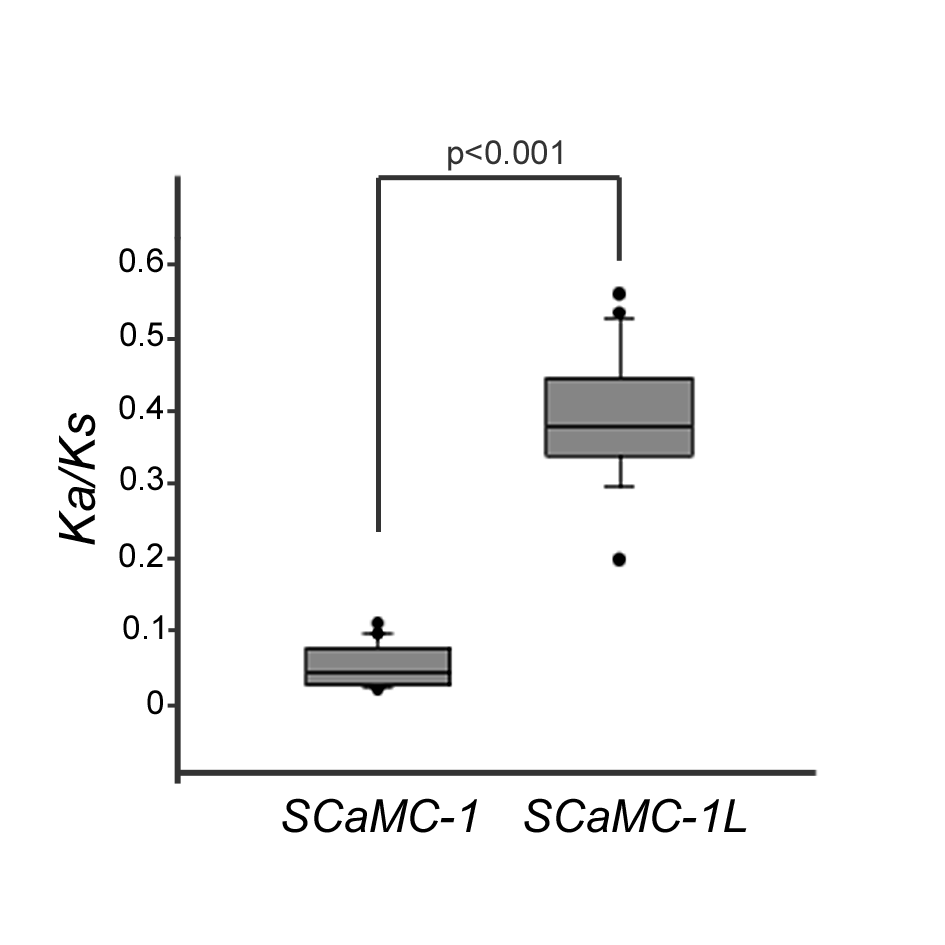

Supplement: Figure S1 — Ka/Ks ratios among mammalian SCaMC-1 and SCaMC-1L orthologues. To calculate Ka/Ks values for SCaMC-1 and SCaMC-1L genes we retrieved coding sequences corresponding to exons 2 to 7 of representative mammalian and performed pairwise sequence comparisons. The results are based on Ka/Ks values for sequences from seven representative mammals; Bos Taurus, Echinops telfairi, Myotis lucifugus, Mus musculus, Macaca mulatta, Pongo pygmaeus, and Rattus novergicus. Ka/Ks ratios were estimated using PAML for all pairwise combinations. The pairwise Ka/Ks ratios for SCaMC-1L orthologs are significantly greater than those of SCaMC-1 (paired t-test; p<0.001) suggesting that SCaMC-1L genes evolved faster than SCaMC-1. (TIF) [file pone.0040470.s001.tif]

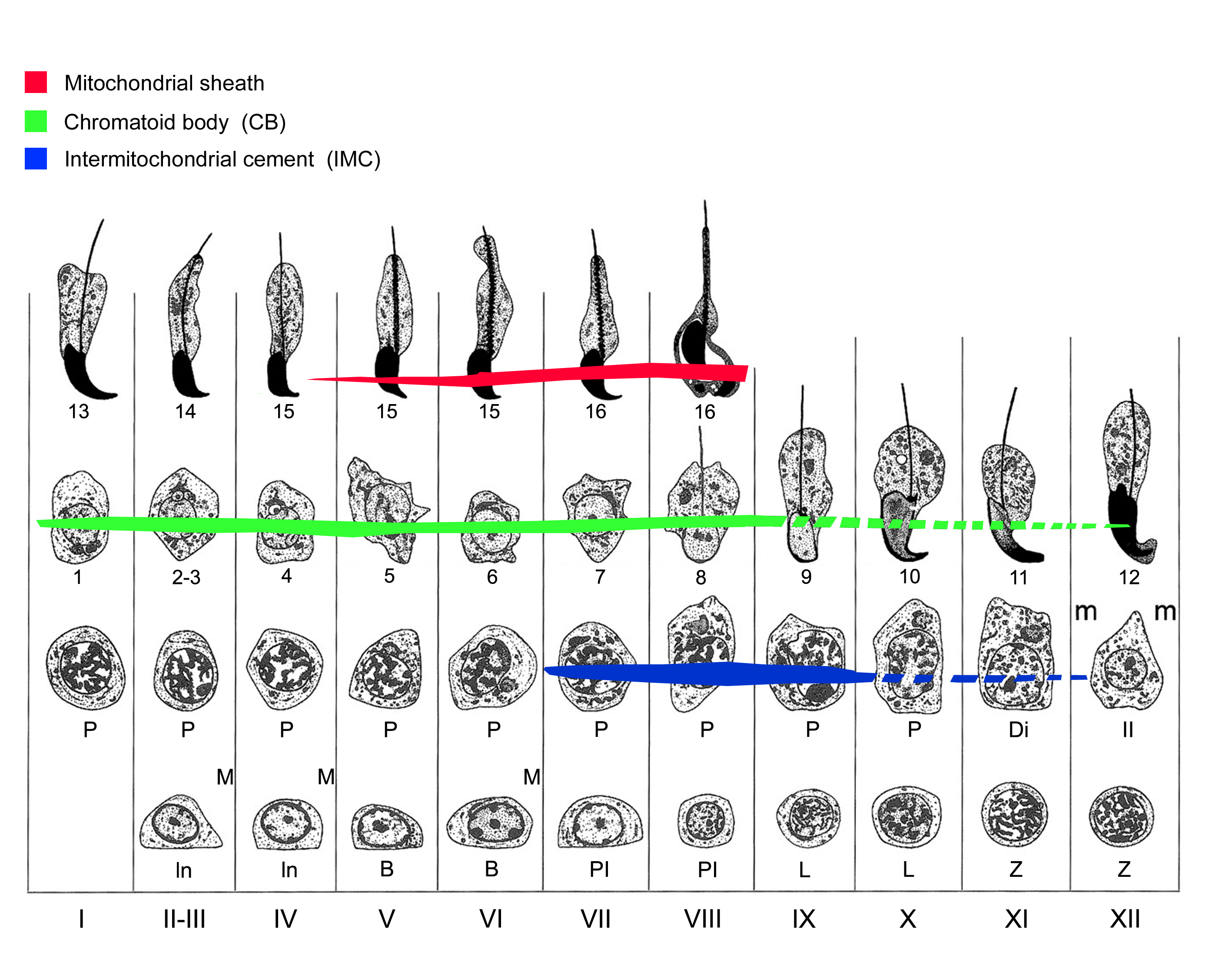

Supplement: Figure S2 — Expression of SCaMC-1L throughout the spermatogenic stages. The germ cells present at each stage (I–XII) of the mouse spermatogenic cycle from intermediate spermatogonia (In) to mature sperm are pictured (adapted from [37]). Each stage of the spermatogenic cycle comprises a set of developing germ cells. In the diagram, the developmental progression of a cell is followed from the bottom file, left to right. By solid colored bars are indicated the developmental stages showing significant SCaMC-1L expression, dashed bars indicate less abundant but detectable expression. The intracellular patterns detected for SCaMC-1L; intermitochondrial cement (IMC), chromatoid body (CB) and mitochondrial sheath, are marked by different colors. SCaMC-1L expression is absent in intermediate and type-B spermatogonia (B), and preleptotene (Pl), leptotene (L) and zygotene spermatocytes (Z). SCaMC-1L is observed in the IMC in late pachytene spermatocytes (P), at differentiation stage VII. After meiosis (m), SCaMC-1L is detected in the CB during round spermatid differentiation (steps 1–8 of spermiogenesis) and as dispersed structures after CB dissociation (step 9 onwards). In elongated spermatids, steps 15 and 16 of spermiogenesis, the protein is found in the mitochondrial sheath of the flagellum. (TIF) [file pone.0040470.s002.tif]

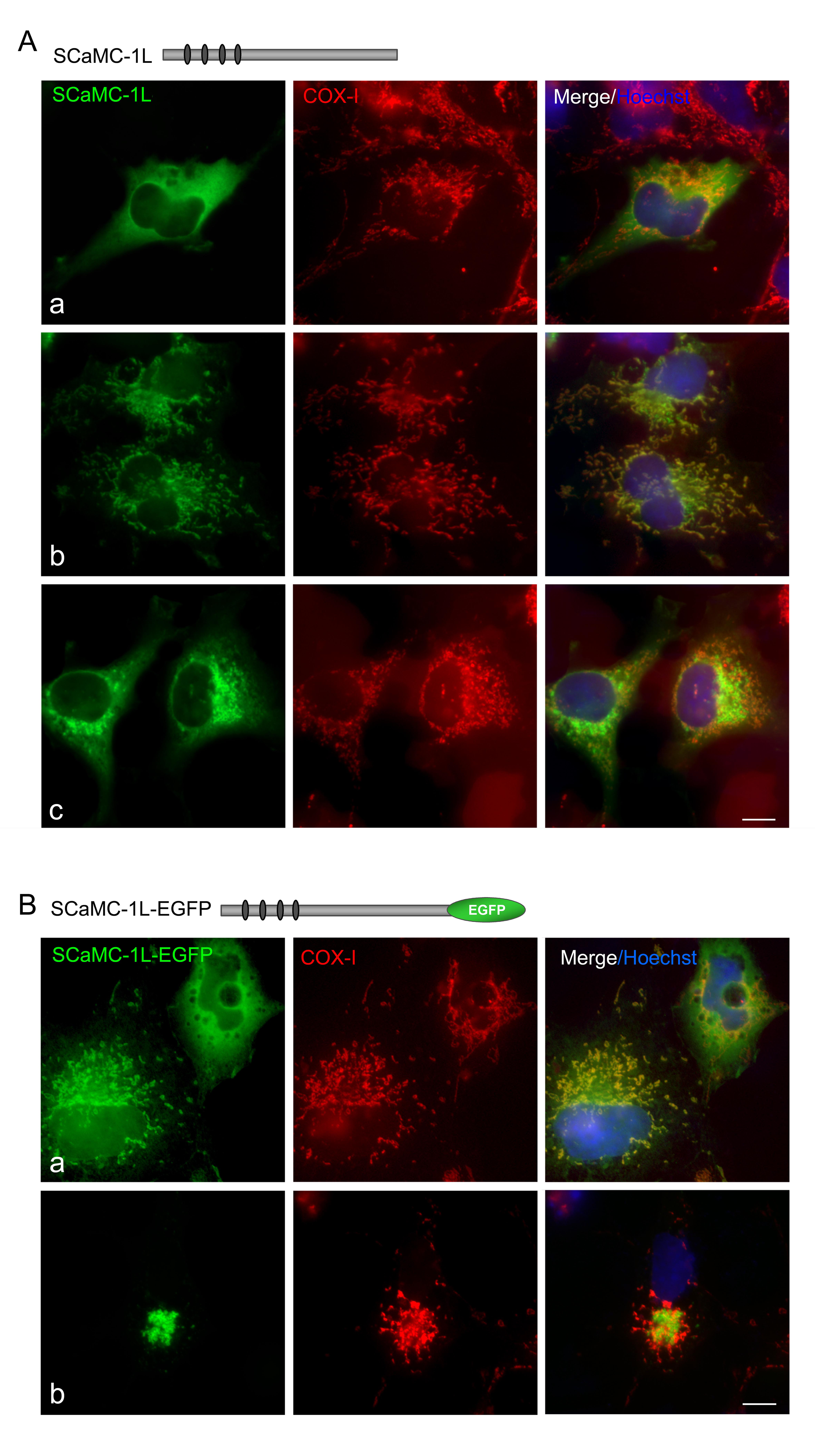

Supplement: Figure S3 — The C-terminal does not affect the intracellular patterns of ScaMC-1L. (A) Representative images of SCaMC-1L-expressing COS-7 cells showing the different intracellular patterns observed; cytosolic (a), mitochondrial (b) and extra-mitochondrial aggregates (c). COS-7 cells were transfected with a full-length SCaMC-1L construct unchanged at C-end, co-localization with mitochondrial structures was determined by co-staining with anti-SCaMC-1L and anti-COX-I as mitochondrial control. (B) Representative images of COS-7 cells expressing a SCaMC-1L-EGFP fusion protein. SCaMC-1L-EGFP construct was obtained subcloning the entire mouse SCaMC-1L coding sequence (amino acids 1–473) into pEGFP-N1. SCaMC-1L-EGFP expressing cells were fixed and co-localization with mitochondrial COX-I was determined. Cells showing cytosolic (a), mitochondrial (a) and extra-mitochondrial aggregates (b) are shown. Magnification 63×; scale bar, 10 µm. (TIF) [file pone.0040470.s003.tif]

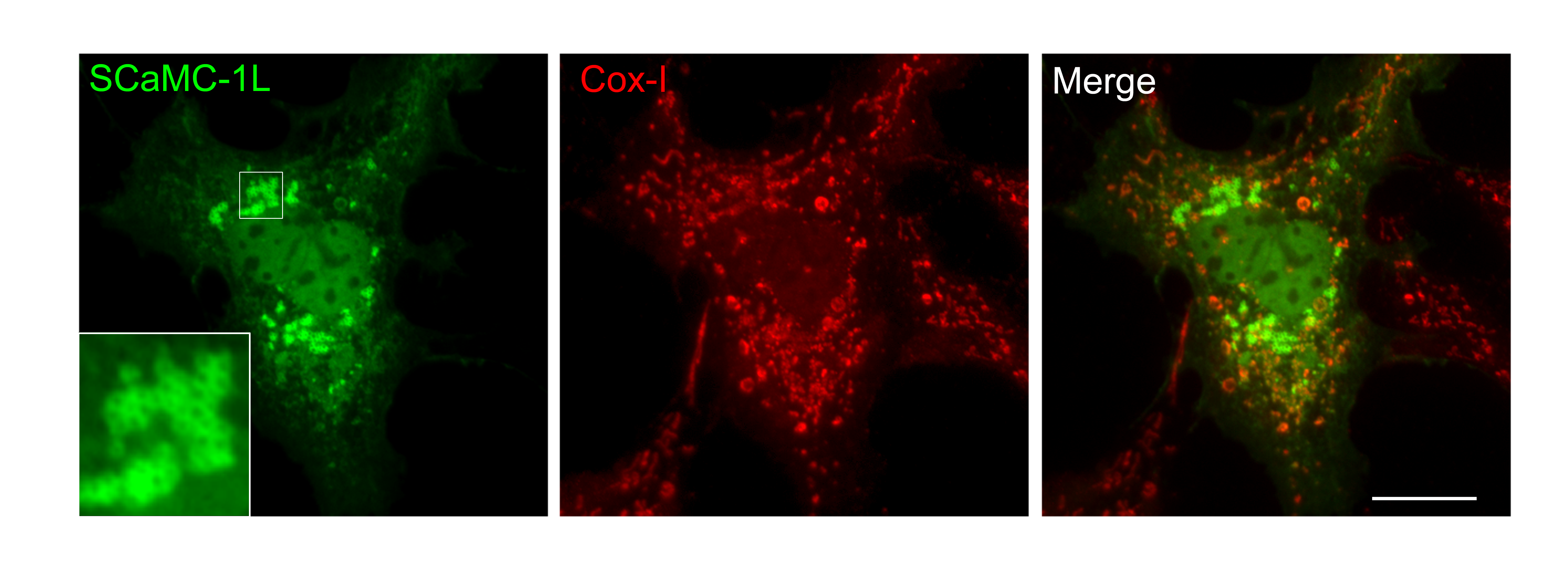

Supplement: Figure S4 — Perinuclear honeycomb-like SCaMC-1L aggregates. A representative image of SCaMC-1L-transfected COS-7 cells containing SCaMC-1L honeycomb-like aggregates is shown. SCaMC-1L was detected with specific anti-SCaMC-1L antibody visualized with a FITC-conjugated secondary antibody, mitochondrial structures are stained with anti-COX-I antibody visualized with Cy3-conjugated secondary antibody; the corresponding merged panel is also shown. Magnification 63×; scale bar, 20 µm. Enlarged image (400×) of the indicated inset is also shown. (TIF) [file pone.0040470.s004.tif]

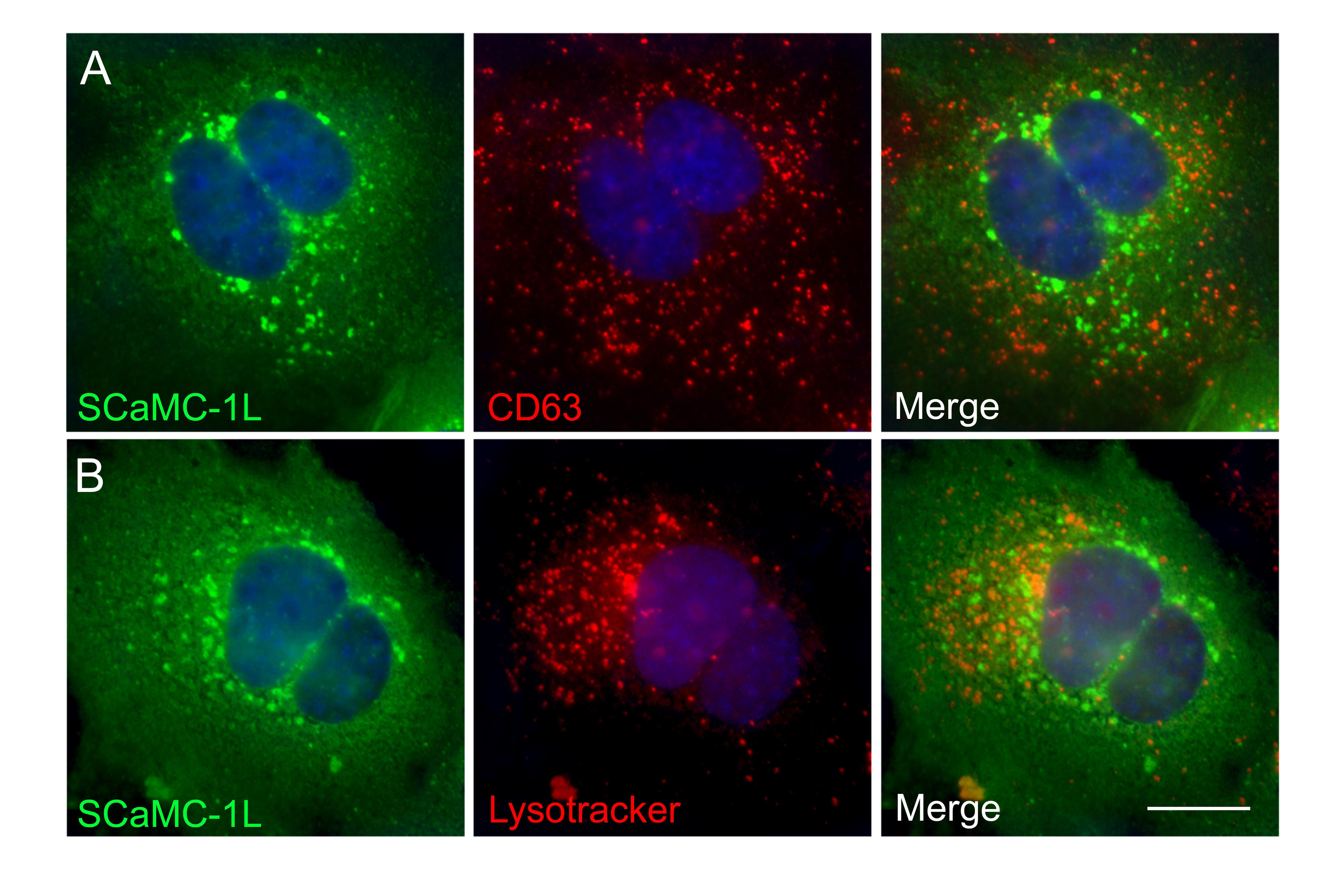

Supplement: Figure S5 — Cytosolic SCaMC-1L granules do not co-localize with late endosomal/lysosomal markers. COS-7 cells were transiently transfected with FLAG-tagged SCaMC-1L and 24–30 hours later co-localization was analyzed by co-staining with specific markers. COS-7 cells were co-stained with anti-SCaMC-1L antibody (A, B) and specific markers for late endosomes (A) and lysosomes (B). As late endosomal marker a monoclonal anti-human CD63 antibody (clone H5C6, Developmental Studies Hybridoma Bank) was used at 1∶200. To analyze co-localization with lysosomes, cells were stained with 50 mM LysoTracker (Molecular Probes) for 30 min at 37°C prior to fixation. Nuclei were stained with Hoechst. Magnification 63×; scale bar, 20 µm. (TIF) [file pone.0040470.s005.tif]

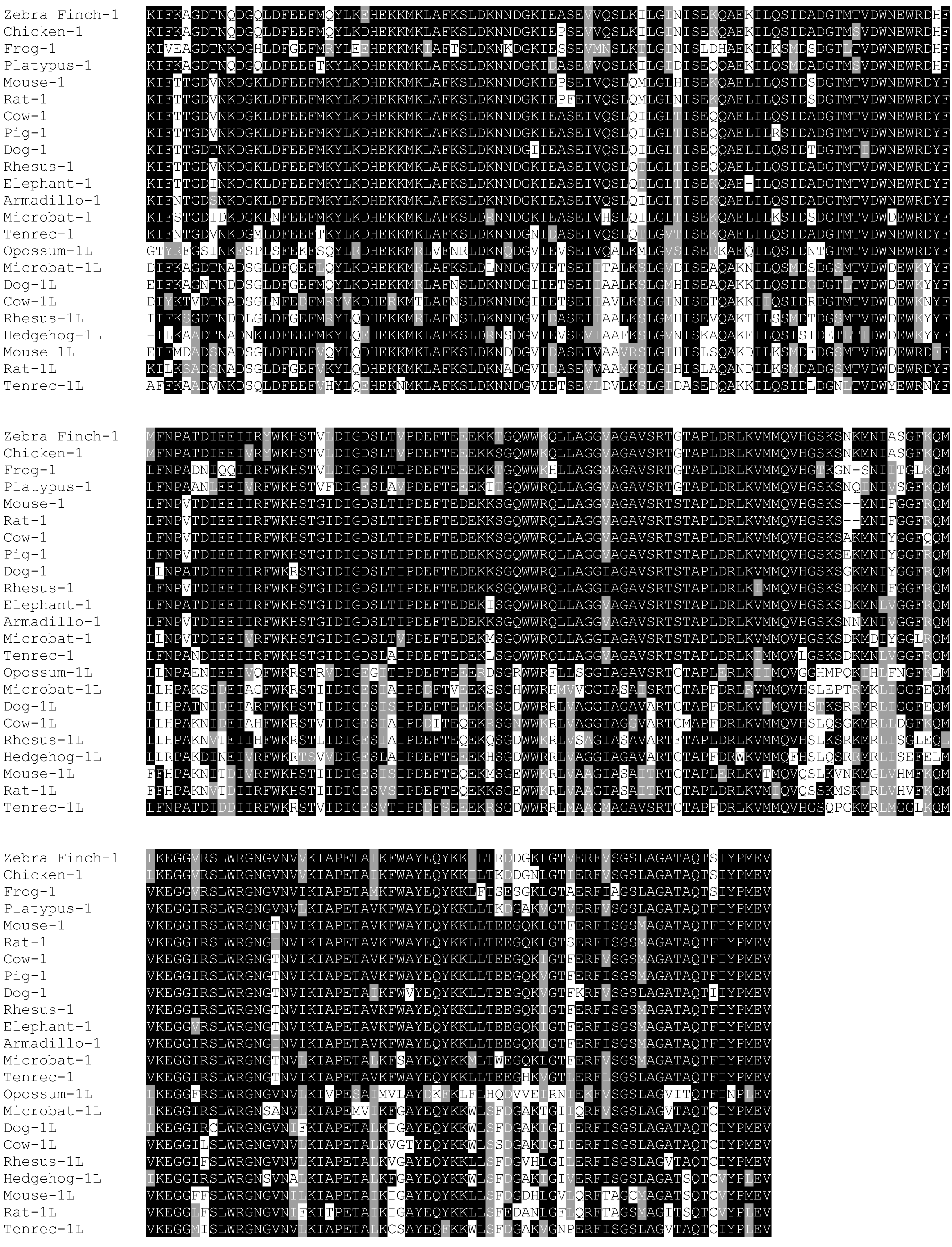

Supplement: Figure S6 — Alignment of SCaMC-1 and SCaMC-1L sequences encoded by exons 2 to 7 was performed with ClustalW program and coloured using BOXSHADE program. (www.ch.embnet.org). (TIF) [file pone.0040470.s006.tif]
