# Supplementary material for: SCaMC-1Like a Member of the Mitochondrial Carrier (MC) Family Preferentially Expressed in Testis and Localized in Mitochondria and Chromatoid Body
Source: PLoS One. 2012 Jul 6;7(7):e40470. doi: 10.1371/journal.pone.0040470 (PMC3391283; doi:10.1371/journal.pone.0040470)
Supplement: Table S1 — Accession numbers of annotated SCaMC-1 and SCaMC-1L proteins. (alphabetical order of species). (DOCX) [file pone.0040470.s007.docx]

**Table SI.** Accession numbers of annotated SCaMC-1 and SCaMC-1L proteins (alphabetical order of species).

| **Specie** | **SCaMC-1** | **SCaMC-1Like** |
| --- | --- | --- |
| *Bos taurus* (cow) | [NP_001092536.1](http://www.ncbi.nlm.nih.gov/entrez/query.fcgi?cmd=Retrieve&db=Protein&list_uids=149642721&dopt=GenPept&RID=7V9F27PM01N&log$=protalign&blast_rank=1) | XP_001789552.1 |
| *Canis familiaris* (dog) | XP_854738.1 | XP_854731 |
| *Dasypus novemcinctus* (armadillo) | [ENSDNOP00000000550](http://www.ensembl.org/Dasypus_novemcinctus/Transcript/ProteinSummary?db=core;g=ENSDNOG00000000725;h=BLAST_NEW:BLA_OnUh66gZ1%21%2120090809;r=GeneScaffold_829:169865-174047;t=ENSDNOT00000000726) |  |
| *Gallus gallus* (chicken) | XP_422180 |  |
| *Homo sapiens* (human) | NP_037518.3 |  |
| *Macaca mulatta* (rhesus monkey) | XP_001083784.1 | XP_001084129.1 |
| *Monodelphis domestica* (opossum) | XP_001381917.1 |  |
| *Mus musculus* (mouse) | BAE41903.1 | [NP_083330.1](http://www.ncbi.nlm.nih.gov/entrez/query.fcgi?cmd=Retrieve&db=Protein&list_uids=198278549&dopt=GenPept&RID=7V9VPNTJ01S&log$=protalign&blast_rank=1) |
| *Rattus novergicus* (rat) | NP_001121016.1 | NP_001103110.1 |
| *Sus scrofa* (pig) | [ACC93575.1](http://www.ncbi.nlm.nih.gov/entrez/query.fcgi?cmd=Retrieve&db=Protein&list_uids=149642721&dopt=GenPept&RID=7V9F27PM01N&log$=protalign&blast_rank=1) |  |
| *Taeniopygia guttata* (zebra finch) | XP_002195432 |  |
| *Xenopus laevis* (frog) | AAH43993.1 |  |
