# Supplementary material for: SCaMC-1Like a Member of the Mitochondrial Carrier (MC) Family Preferentially Expressed in Testis and Localized in Mitochondria and Chromatoid Body
Source: PLoS One. 2012 Jul 6;7(7):e40470. doi: 10.1371/journal.pone.0040470 (PMC3391283; doi:10.1371/journal.pone.0040470)
Supplement: Table S2 — SCaMC-1 and SCaMC-1L non-annotated protein sequences used in this study. (alphabetical order of species). (DOCX) [file pone.0040470.s008.docx]

**Table SII**. Sequence of SCaMC-1 and SCaMC-1L non-annotated proteins used in this study (alphabetical order of species)

| **Species** | **Proteins^1^** |
| --- | --- |
| *Erinaceus europaeus* (hedgehog) | **>SCaMC-1Like** (partial sequence)  AILKAADTNADNKLDFEEFMKYLQEHEKKMKLAFKSLDRNSDGVIEVSEVIAAFKSLGVNISKAQAKEILQSISIDETLTIDWDEWKYYFLLRPAKDINEIVRFWKRTSVVDIGESLAIPDEFTEEEKHSGDWWRRLVAGGIAGAVARTCTAPFDRWKVMMQFHSLQSRRMRLISEFELMIKEGGIRSLWRGNSVNALKIAPETALKFGAYEQYKKWLSFDGAKIGIVERFISGSLAGATSQTCVYPLEVLKTRLAISTTGQYTGAIDCAKKILKQEGVRVFFKGYVPNLLGIIPYAGIDLAVYELLKNYWLEHHAEDSLNPGIMILLAFSTFSHTCGQVVSFPLNLIRTHMQAKALEESPPSMIDFIQDVYNKEGARGFFRGLTPNIIKVLPAVIISCVTFEKVKNYLGFI |
| *Echinops telfairi* (lesser hedgehog tenrec) | **>SCaMC-1**  MLRWLGTLVLPMAACQDEQQPTRYQSLFQQLDRNRDGVVDIGELQQGLRSLGVPLGQDAEEKIFNTGDVNKDGMLDFEEFTKYLKDHEKKMKLAFKSLDKNNDGNIDASEIVQSLQTLGVTISEKQAELILQSIDADGTMTVDWNEWRDYFLFNPANDIEEIIRFWKHSTGIDIGDSLAIPDEFTEDEKLSGQWWRQLLAGGVAGAVSRTSTAPLDRLKIMMQVLGSKSDKMNLVGGFRQMVKEGGIRSLWRGNGTNVIKIAPETAVKFWAYEQYKKLLTEEGHKVGTLERFLSGSLAGATAQTFIYPMEVFKTRLAVAKTGQYSGIFDCAKKILKHEGMGALYKGYIPNLLGIISYAGIDLAVYELLKSYWLEHFAEDTVNPGVVVLLGCGALSSTCGQLASYPLALVRTRMQAQAMVDGAPQLNMVGLFRRIISKEGARGLYRGITPNFMKVLPAVGISYVVYEKMKQTLGVAKK  **>SCaMC-1Like** (partial sequence)  AFFKAADVNKDSQLDFEEFVHYLQEHEKNMKLAFKSLDKNNDGVIETSEVLDVLKSLGIDASEDQAKKILQSIDLDGNLTVDWYEWRNYFLFNPATDIDDIIRFWKRSTVIDIGESVTIPDDFSEEEKRSGDWWRRLMAAGMAGAVSRTCTAPFDRLKVMMQVHGSQPGKMRLMGGLKQMVKEGGMISLWRGNGVNVLKIAPETALKCSAYEQFKKWLSFDGAKVGNPERFISGSLAGVTAQTCIYPLEVLKTRLAVGQTGQYSGVIDCGKKLLKQEGVKALFKGYFPNVLGIIPYAGIDFAVFELLKNYWLENHAKNSVNPGIMILLGCCTLSSTCGQLASFPLHLLRTRLQAQAHVNGGSITSMIHLIQEIYHKEGKRGFFRGLTPNIIKVLPAVGISCLVYENVKPFVGL |
| *Microcebus murinus* (grey mouse lemur) | **>SCaMC-1Like** (partial sequence)  VIFRAGDTNADSKLDFGEFLRYLQDHEKKMRLAFNSLDTNKNGVIEVSEILAALKSMGMNISEEQAVEIIKSMDTDGTMTIDWDEWKYYFLLHPATNITEIIHFWKHSAVIDIGESISIPDDFTEHEKQSGDWWKRLVAAGIASAIARTCTAPFERLRVMLQVHSLKTRKMRLTSVVEQMIKEGGIFSLWRGNGVNIFKIAPETALKVGAYEQYKKWLSFDGTHVGIPERFISGSLAGVTAQTCIYPMEVLKTRLAVGNTGEYSGIIDCSRKLFKQEGVRAFFRGYVPNFLGIIPYAGIDLAVYELLKNYWLEHYSKNSVNPGIMILLGCSTLSHTCGQLASFPLNLLRTRMQAKAPMEEGKTVSMIRLIQEIYSKEGKRGFFRGITPNVIKLLPAVGISCVAYEKVKLLMGLT |
| *Myotis lucifugus* (Microbat) | **>SCaMC-1**  MLRWLRGWVLPAAACQDAGPPPPRYETLFRQLDRNGDGVVDIGELQEGLRSLGIPLGQDAEEKIFSTGDIDKDGKLNFEEFMKYLKDHEKKMKLAFKSLDRNNDGKIEASEIVHSLQILGLTISEKQAELILKSIDSDGTMTVDWDEWRDYFLLNPVTDIEEIVRFWKHSTGIDIGDSLTVPDEFTEDEKMSGQWWRQLLAGGIAGAVSRTSTAPLDRLKVMMQVHGSKSDKMDIYGGLRQMVKEGGIRSLWRGNGTNVLKIAPETALKFSAYEQYKKMLTWEGQKLGTFERFVSGSMAGATAQTFIYPMEVLKTRLAVGRTGQYSGLFDCAKKILKHEGMGAFFKGYIPNILGIIPYAGIDLAVYELLKSHWLDHFAKDTVNPGVAVLLGCGALSSTCGQLASYPLSLVRTRMQAQAMIEGSPQLNMVGLFRRIISKEGVPGLYRGITPNFMKVLPAVGISYVVYENMKQTLGVIQK  **>SCaMC-1Like** (partial sequence)  DIFKAGDTNADSGLDFQEFLQYLKDHEKKMRLAFKSLDLNNDGVIETSEIITALKSLGVDISEAQAKNILQSMDSDGSMTVDWDEWKYYFLLHPAKSIDEIAGFWKRSTIIDIGESIAIPDDFTVEEKSSGHWWRHMVVGGIASAISRTCTAPFDRLRVMMQVHSLEPTRMKLIGGFEQMIKEGGIRSLWRGNSANVLKIAPEMVIKFGAYEQYKKWLSFDGAKTGIIQRFVSGSLAGVTAQTCIYPMEVIKTRLTVGRTGQYSGIIDCGKKLLKQEGVRTFFKGYVPNLLSIIPYAGTDLTVFELLKNYWLEHYAGSSVDPGLMILLGCSTLSHTSGQIASFPLTLLRTRMQAQAQKEKTTTMIHLIQDIYYKEGKMGFFRGLTPNIIKVLPAIFISCVAYEILKRPFGLT |
| *Ornithorhynchus anatinus* (duck-billed platypus) | **>SCaMC-1** (Partial sequence assembled from UCSC Contig4142 and Contig243411)  KIFKAGDTNQDGQLDFEEFTKYLKDHEKKMKLAFKSLDKNNDGKIDASEVVQSLKILGIDISEQQAEKILQSMDADGTMSVDWNEWRDHFLFNPAANLEEIVRFWKHSTVFDIGESLAVPDEFTEEEKTTGQWWRQLLAGGVAGAVSRTGTAPLDRLKVMMQVHGSKSNQINIVSGFKQMVKEGGIRSLWRGNGVNVLKIAPETAVKFWAYEQYKKLLTKDGAKVGTVERFVSGSLAGATAQTFIYPME |
